# Radiomics for therapy-specific head and neck squamous cell carcinoma survival prognostication (part I)

**DOI:** 10.1186/s12880-023-01034-1

**Published:** 2023-06-02

**Authors:** Simon Bernatz, Ines Böth, Jörg Ackermann, Iris Burck, Scherwin Mahmoudi, Lukas Lenga, Simon S. Martin, Jan-Erik Scholtz, Vitali Koch, Leon D. Grünewald, Ina Koch, Timo Stöver, Peter J. Wild, Ria Winkelmann, Thomas J. Vogl, Daniel Pinto dos Santos

**Affiliations:** 1 Department of Diagnostic and Interventional Radiology, University Hospital Frankfurt, Goethe University Frankfurt Am Main, Theodor-Stern-Kai 7, Frankfurt Am Main, 60590 Germany; 2 Dr. Senckenberg Institute for Pathology, University Hospital Frankfurt, Goethe University Frankfurt Am Main, Frankfurt Am Main, 60590 Germany; 3 Frankfurt Cancer Institute (FCI), Frankfurt Am Main, 60590 Germany; 4 Department of Molecular Bioinformatics, Institute of Computer Science, Johann Wolfgang Goethe-University, Robert-Mayer-Str. 11-15, Frankfurt Am Main, 60325 Germany; 5 Department of Otorhinolaryngology, University Hospital Frankfurt, Goethe University Frankfurt Am Main, Theodor-Stern-Kai 7, Frankfurt Am Main, 60590 Germany; 6 Frankfurt Institute for Advanced Studies (FIAS), Frankfurt Am Main, 60438 Germany; 7 Department of Diagnostic and Interventional Radiology, University of Cologne, Faculty of Medicine and University Hospital Cologne, Kerpener Str. 62, Cologne, 50937 Germany

**Keywords:** Medical imaging, Survival prediction, Radiomics, Machine learning, Artificial intelligence

## Abstract

**Background:**

Treatment plans for squamous cell carcinoma of the head and neck (SCCHN) are individually decided in tumor board meetings but some treatment decision-steps lack objective prognostic estimates. Our purpose was to explore the potential of radiomics for SCCHN therapy-specific survival prognostication and to increase the models’ interpretability by ranking the features based on their predictive importance.

**Methods:**

We included 157 SCCHN patients (male, 119; female, 38; mean age, 64.39 ± 10.71 years) with baseline head and neck CT between 09/2014 and 08/2020 in this retrospective study. Patients were stratified according to their treatment. Using independent training and test datasets with cross-validation and 100 iterations, we identified, ranked and inter-correlated prognostic signatures using elastic net (EN) and random survival forest (RSF). We benchmarked the models against clinical parameters. Inter-reader variation was analyzed using intraclass-correlation coefficients (ICC).

**Results:**

EN and RSF achieved top prognostication performances of AUC = 0.795 (95% CI 0.767–0.822) and AUC = 0.811 (95% CI 0.782–0.839). RSF prognostication slightly outperformed the EN for the complete (ΔAUC 0.035, *p* = 0.002) and radiochemotherapy (ΔAUC 0.092, *p* < 0.001) cohort. RSF was superior to most clinical benchmarking (*p* ≤ 0.006). The inter-reader correlation was moderate or high for all features classes (ICC ≥ 0.77 (± 0.19)). Shape features had the highest prognostic importance, followed by texture features.

**Conclusions:**

EN and RSF built on radiomics features may be used for survival prognostication. The prognostically leading features may vary between treatment subgroups. This warrants further validation to potentially aid clinical treatment decision making in the future.

**Supplementary Information:**

The online version contains supplementary material available at 10.1186/s12880-023-01034-1.

## Background

Squamous cell carcinoma of the head and neck (SCCHN) is among the most common cancers worldwide [1] with a 5-year relative survival of 52.1% [2]. At baseline, the primary tumor and lymph nodes are assessed by contrast-enhanced computed tomography (CT) scans and/or magnetic resonance imaging [3]. The clinical workup for staging and diagnosis further includes, for example, pathological confirmation, general clinical examinations as well as the strong recommendation to perform head and neck endoscopy and fluorodeoxyglucose-positron emission tomography [3]. The TNM Classification of Malignant Tumors (TNM) is based on the three alphanumeric codes T, N, and M to describe the primary tumor, regional lymph nodes, and metastasis, respectively and it is a known prognostic factor for patient survival based on disease stage [3]. Pre-treatment risk assessment is the cornerstone for effective treatment planning to achieve the best cure rates and lowest risk of morbidity [3]. Therefore, treatment plans are based on tumor (e.g. TNM stage) as well as patient (e.g. age) characteristics [3]. Treatment regimens are complex, i.e. single-modality treatment or variant combinations of surgery, radiation, chemotherapy and systemic therapy with and without potential optional steps [3]. The final treatment plan is a consensus finding of a multidisciplinary team including various treatment disciplines (e.g. surgery, radiation oncology), diagnosis (e.g. radiology) and treatment support [3]. Two biomarkers are currently captured in the clinical practice guidelines [3] with prognostic (p16) or therapeutic (programmed death-ligand 1) value [3]. Recent advances in CT and magnetic resonance imaging technologies, quantitative imaging biomarkers and artificial intelligence provide promising opportunities, especially in oncology [4]. CT and MRI may yield complementary information; i.e. MRI may allow for quantitative diffusion imaging but CT measures tend to be more stable which facilitates their implementation in artificial intelligence modeling [3, 5, 6]. Radiomics describes the transformation of images into mineable data and it has the potential to characterize tumor characteristics beyond visual perception [7]. Radiomics biomarkers have shown promising results in characterizing different tumor types [8, 9] but there is no evidence yet in treatment-specific survival prognostication of SCCHN.

We hypothesize that imaging biomarkers offer treatment-specific prognostic capabilities in survival prediction. Therefore, the goal of this study was to analyze the primary SCCHN tumor by means of radiomics features to evaluate the treatment-specific model performance and treatment-specific feature importance for continuous survival prognostication. Furthermore, analysis and ranking of imaging features according to their predictive importance was carried out to improve the interpretability of the model.

## Methods

The local ethics committee of the Goethe University Frankfurt am Main, Germany approved this retrospective study (project number: 20–890) and waived informed written consent.

### Study design

**Fig. 1:**
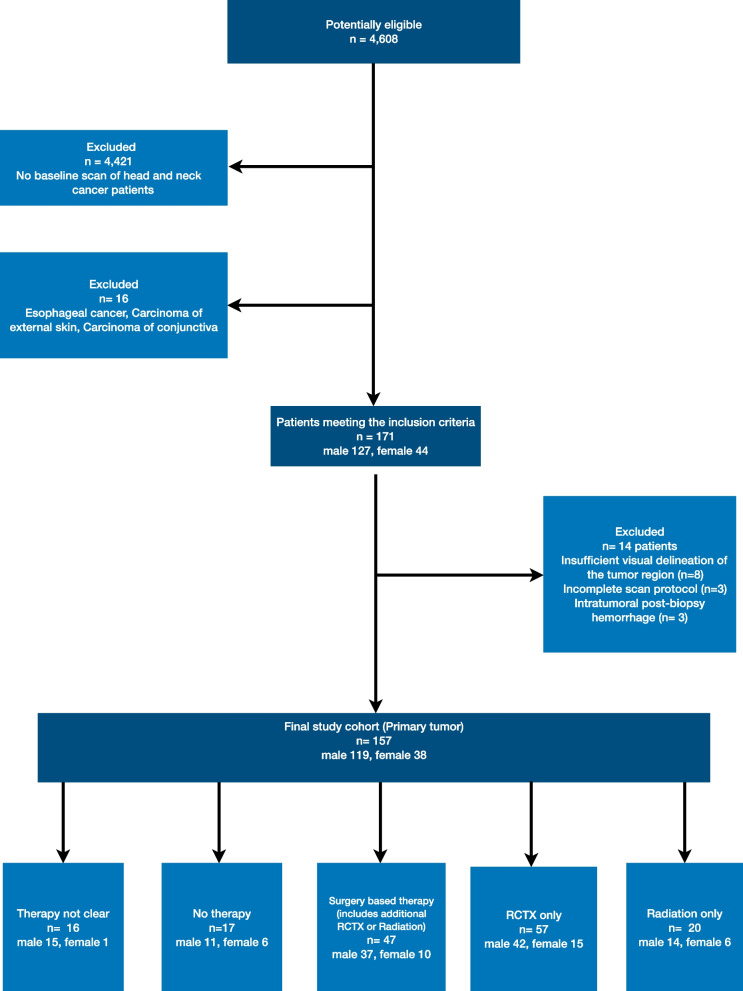
STARD Flowchart of patient inclusion into the study. The study cohort comprised 157 patients which were enrolled into the respective subgroups as depicted. RCTX, radiochemotherapy

We screened a total of 4,608 consecutive patients for study inclusion. The screening cohort comprised patients who underwent contrast-enhanced CT imaging of the head and neck between 09/2014 and 08/2020 on one single CT system. Further inclusion criteria were (I) > 18 years of age, (II) 2 mm axial plane reconstruction, (III) baseline pretherapeutic imaging, (IV) histological confirmation of SCCHN (oral cavity, pharynx, larynx, nasal/sinus/nasopharyngeal). Exclusion criteria were (I) imaging artefacts affecting the tumor region, (II) insufficient visual delineation of the tumor, (III) post-biopsy hemorrhage in the tumor, (IV) incomplete scan protocol. Consequently, 157 patients (male, 119; female, 38; mean age, 64.39 ± 10.71 years) were evaluated. In Fig. 1 we depict the STARD Flowchart of study inclusion and indicate the number of excluded patients for each cause. In Table 1 we summarize the clinical and epidemiological characteristics.

**Table 1:** Demographics, clinical and epidemiological characteristics of included patients

	**Complete cohort**	**Surgery cohort**	**RCTX cohort**	**Radiation cohort**	**No therapy**	**Therapy unclear **
**Patients**	**157 (100%)**	**47 (29.94%)**	**57 (36.31%)**	**20 (12.74%)**	**17 (10.83%)**	**16 (10.19%)**
Male	119 (75.8%)	37 (78.72%)	42 (73.68%)	14 (70%)	11 (64.7%)	15 (93.75%)
Female	38 (24.2%)	10 (21.28%)	15 (26.32%)	6 (30%)	6 (35.29%)	1 (6.25%)
Age at initial diagnosis (mean, std, years)	64.39 (+/- 10.71)	61.74 (+/- 9.24)	61.6 (+/- 9.49)	71.6 (+/- 12.14)	70.6 (+/- 10.86)	63.06 (+/- 11.86)
**Tumor localization**						
Outer nose and nasal cavities	8 (5.09%)	2 (4.25%)	2 (3.51%)	2 (10%)	0 (0%)	2 (12.5%)
Oral cavity	36 (22.92%)	18 (38.3%)	5 (8.77%)	4 (20%)	5 (29.41%)	4 (25%)
Tounge margin	1 (0.64%)	0 (0%)	0 (0%)	0 (0%)	1 (5.88%)	0 (0%)
Oropharynx	51 (32.48%)	9 (19.15%)	23 (40.35%)	7 (35%)	7 (41.18%)	5 (31.25%)
Hypopharynx	14 (8.92%)	1 (2.13%)	8 (14.04%)	3 (15%)	0 (0%)	2 (12.5%)
Larynx	47 (29.94%)	17 (36.17%)	19 (33.33%)	4 (20%)	4 (23.53%)	3 (18.75%)
**Carcinogen exposure**	**52** [105] (33.12%)	**15** [32] (31.91%)	**19** [38] (33.33%)	**10** [10] (50%)	**4** [13] (23.53%)	**4** [12] (25%)
Alcohol	3 [105] (5.77%)	1 [32] (6.67%)	1 [38] (5.26%)	0 [10] (0%)	1 [13] (25%)	0 [12] (0%)
Smoking	26 [105] (50%)	7 [32] (46.67%)	9 [38] (47.37%)	6 [10] (60%)	2 [13] (50%)	2 [12] (50%)
Alcohol *and* smoking	23 [105] (44.23%)	7 [32] (46.67%)	9 [38] (47.36%)	4 [10] (40%)	1 [13] (50%)	2 [12] (50%)
**Immunohistochemistry at initial diagnosis**^**ab**^						
***CK 5/6***^**b**^*** and p63***^**b**^*** tested***	**99** [58] (63.06%)	**30** [17] (63.83%)	**39** [18] (68.42%)	**14** [6] (70%)	**11** [6] (64.71%)	**5** [11] (31.25%)
CK 5/6^b^ and p63^b^ positive	84 [58] (84.85%)	26 [17] (86.67%)	31 [18] (79.5%)	13 [6] (92.9%)	10 [6] (90.9%)	4 [11] (80%)
CK 5/6^b^ and p63^b^ negative	15 [58] (15.15%)	4 [17] (13.33%)	8 [18] (20.5%)	1 [6] (7.1%)	1 [6] (9.1%)	1 [11] (20%)
***p16***^**b**^*** tested***	**99** [58] (63.06%)	**30** [17] (63.83%)	**39** [18] (68.42%)	**14** [6] (70%)	**11** [6] (64.71%)	**5** [11] (31.25%)
p16^b^ positive	27 [58] (27.27%)	6 [17] (20%)	13 [18] (33.3%)	3 [6] (21.43%)	2 [6] (18.2%)	3 [11] (60%)
p16^b^ negative	72 [58] (72.73%)	24 [17] (80%)	26 [18] (66.7%)	11 [6] (78.57%)	9 [6] (81.8%)	2 [11] (40%)
**Grading**^**c**^						
CIS (carcinoma in situ)	6 [1] (3.85%)	0 (0%)	3 [1] (5.36%)	1 (5%)	0 (0%)	2 (12.5%)
G1	5 [1] (3.21%)	3 (6.4%)	0 [1] (0%)	1 (5%)	1 (5.88%)	0 (0%)
G2	118 [1] (75.64%)	37 (78.2%)	41 [1] (73.21%)	16 (80%)	13 (76.47%)	11 (68.75%)
G3	24 [1] (15.38%)	7 (14.9%)	10 [1] (17.86%)	1 (5%)	3 (17.65%)	3 (18.75%)
No grading provided^d^	3 [1] (1.92%)	0 (0%)	2 [1] (3.57%)	1 (5%)	0 (0%)	0 (0%)
**TNM-Staging**						
***cTNM-Staging ***						
cT1	19 (12.10%)	15 (31.91%)	2 (3.51%)	0 (0%)	1 (5.88%)	1 (6.25%)
cT2	29 (18.47%)	11 (23.4%)	12 (21.05%)	4 (20%)	0 (0%)	2 (12.5%)
cT3	32 (20.38%)	6 (14.63%)	12 (21.05%)	5 (25%)	5 (29.41%)	4 (25%)
cT4	77 (49.04%)	15 (31.91%)	31 (54.39%)	11 (55%)	11 (22%)	9 (56.25%)
cN0	52 (33.12%)	24 (51.06%)	10 (17.54%)	8 (40%)	4 (23.53%)	6 (37.5%)
cN1	28 (17.83%)	12 (25.53%)	8 (14.04%)	2 (10%)	3 (17.65%)	3 (18.75%)
cN2	55 (35.03%)	6 (12.77%)	31 (54.39%)	7 (35%)	5 (29.41%)	6 (37.5%)
cN3	4 (2.54%)	0 (0%)	2 (3.51%)	0 (0%)	2 (11.76%)	0 (0%)
cNX	18 (11.46%)	5 [42] (10.64%)	6 (10.53%)	3 (15%)	3 (17.65%)	1 (6.25%)
cM0	148 (94.27%)	47 (100%)	53 (92.98%)	20 (100%)	13 (76.47%)	15 (93.75%)
cM1	5 (3.19%)	0 (0%)	3 (5.26%)	0 (0%)	2 (11.76%)	0 (0%)
cMX	4 (2.54%)	0 (0%)	1 (1.75%)	0 (0%)	2 (11.76%)	1 (6.25%)
***pTNM-Staging***^**e**^	**47 **[110] (29.94%)					
***pT***	**39** [118] (24.84%)	**39** [8] (83%)				
pT0	1 [118] (2.56%)	1 [8] (2.56%)				
pT1	9 [118] (23.08%)	9 [8] (23.08%)				
pT2	13 [118] (33.33%)	13 [8] (33.33%)				
pT3	6 [118] (15.38%)	6 [8] (15.38%)				
pT4	10 [118] (25.64%)	10 [8] (25.64%)				
pTX	0 [118] (0%)	0 [8] (0%)				
pTis	0 [118] (0%)	0 [8] (0%)				
***pN***	**39** [118] (24.84%)	**39** [8] (83%)				
pN0	25 [118] (64.01%)	25 [8] (64.01%)				
pN1	6 [118] (15.38%)	6 [8] (15.38%)				
pN2	6 [118] (15.38%)	6 [8] (15.38%)				
pN3	2 [118] (5.12%)	2 [8] (5.12%)				
pNX	0 [118] (0%)	0 [8] (0%)				
***pM***	**39** [118] (24.84%)	**39** [8] (83%)				
pM0	39 [118] (100%)	39 [8] (100%)				
pM1	0 [118] (0%)	0 [8] (0%)				
***L***	**30** [127] (19.11%)	**30** [17] (63.83%)				
L0	24 [127] (80%)	24 [17] (80%)				
L1	6 [127] (20%)	6 [17] (20%)				
***V***	**31** [126] (19.75%)	**31** [16] (65.96%)				
V0	28 [126] (90.32%)	28 [16] (90.32%)				
V1	3 [126] (9.68%)	3 [16] (9.68%)				
***Pn***	**30** [127] (19.1%)	**30** [17] (63.83%)				
Pn0	27 [127] (90%)	27 [17] (90%)				
Pn1	3 [127] (10%)	3 [17] (10%)				
***Resection margin***	**32** [125] (20.38%)	**32** [15] (68.1%)				
R0	28 [125] (87.5%)	28 [15] (87.5%)				
R1	3 [125] (9.4%)	3 [15] (9.4%)				
RX	1 [125] (3.1%)	1 [15] (3.1%)				
**Local histologic follow-up**	**80** [77] (50.96%)	**28** [19] (59.57%)	**39** [18] (68.42%)	**8** [12] (40%)	**0** [17] (0%)	**5** [11] (31.25%)
Local relapse histologically confirmed	18 [77] (22.5%)	9 [19] (32.14%)	4 [18] (10.26%)	2 [12] (25%%)	0 [17] (0%)	3 [11] (60%)
Local relapse histologically excluded	62 [77] (77.5%)	19 [19] (67.86%)	35 [18] (89.74%)	6 [12] (75%)	0 [17] (0%)	2 [11] (40%)

Values in square brackets indicate non available data. ^a^99 out of a total of 157 patients had an immunhistochemistry at initial diagnosis. Markers were stained on a patient-specific basis in clinical routine. ^b^First introduced 11/2014. ^c^A three-stage grading system was applied in clinical routine. ^d^No grading provided for p16 positive squamous cell carcinoma of the oropharynx. ^∆^Only known in surgically treated patients (*n* = 47). Not every surgically treated patient received a determination of pTNM-, L-, V-, Pn- or R-status as determined in square brackets. Percentage values in round brackets indicate the percentage of the pTNM distribution within the surgery-cohort

### Reference standard

All tumors were histologically confirmed in the institution’s pathology department. The clinical data and tumor stage were extracted from the written reports and the consensus statements of multidisciplinary tumor board meetings. Overall survival was defined as the primary outcome measure.

### CT acquisition and reconstruction

Examinations were performed on a third-generation, dual-source, dual-energy CT system (Somatom Force, Siemens Healthineers, Forchheim, Germany). After the acquisition of a scout, the image acquisition (caudocranial direction) was performed during the venous phase following routine protocols: automatic start 70 s after the beginning of the contrast agent (Imeron 400, Bracco, Milan, Italy) injection (dose: 1.2 mL/kg of body weight, maximum volume: 120 mL, flow-rate: 3 mL/s) through a peripheral vein of the forearm. The X-ray tubes were operated with the settings: tube A, 90 kV, reference current–time product of 90mAs; tube B, Sn150kV [0.64 mm tin filter], 69mAs (reference); rotation time, 0.25 s; pitch, 0.7; collimation, 2 × 128 × 0.6 mm. Attenuation-based tube current modulation (CARE Dose 4D, Siemens) and third-generation advanced modeled iterative reconstruction (ADMIRE, Siemens; strength level 3) with a medium smooth reconstruction kernel (Br40) was used. Images were generated in clinical routine using weighted averages from both detectors (60% low kV, 40% high kV spectrum). For each patient the volume CT dose index and the dose length product was recorded. All acquisitions were reconstructed as axial slices with 2 mm slice thickness in clinical routine. For the radiomics analysis the 2 mm axial images were exported in Digital Imaging and Communications in Medicine (DICOM) format.

### Image preprocessing and segmentation

**Fig. 2:**
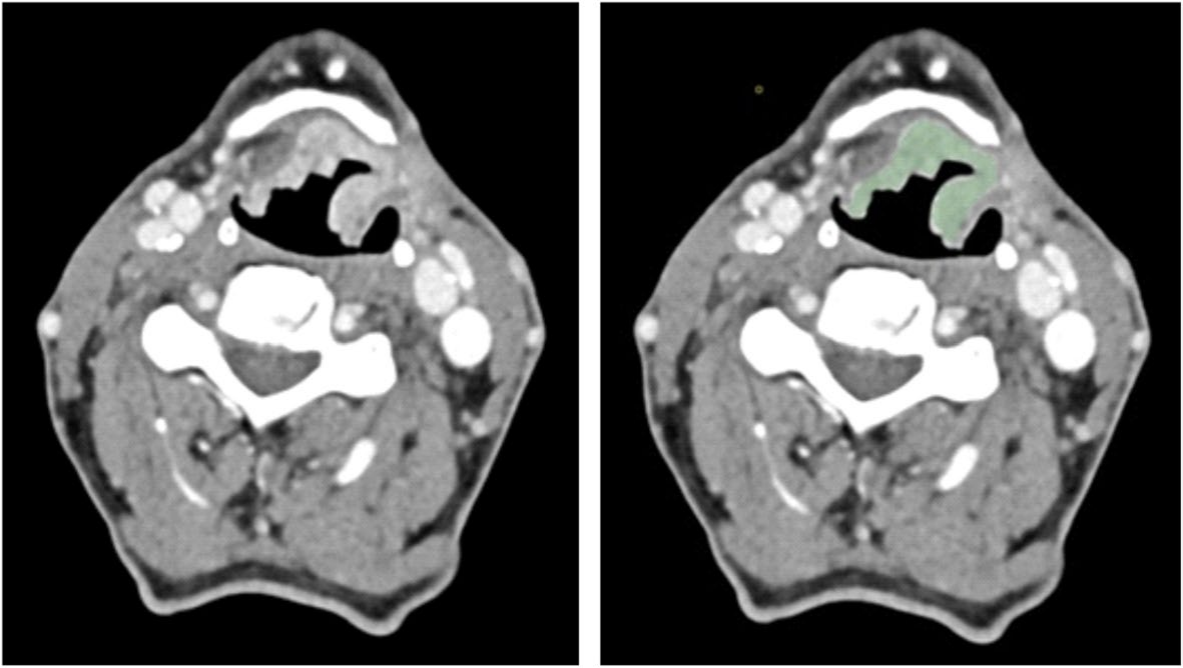
Region of interest circumscription. Computed tomography images of a representative patient to depict the workflow of region of interest definition (ROI). From left to right the original image and radiomics ROI are shown. Patient with T2N0M0 laryngeal squamous cell carcinoma who was treated with definite radiochemotherapy who was still alive at the last documented contact 945 days after the initial diagnosis

For the visualization and processing of the DICOM image stack we used the 3D Slicer software platform (http://slicer.org, version 4.11.20200930) [10]. We resampled the images to a spacing of 1 mm × 1 mm × 1 mm employing B-spline interpolation (https://www.slicer.org/wiki/Registration:Resampling, supplementary methods 2 of Griethuysen et al. [11]) [10]. We did not perform further image manipulation as the Imaging Biomarker Standardization (IBSI) does currently not cover image preprocessing [12]. One especially trained investigator (InB, 1 year of experience), manually delineated each tumor on a representative 2D plane with the biggest tumor area, sparing calcifications and air bubbles for radiomics analysis (Fig. 2). All segmentations were independently reviewed by a radiologist (SB, 3 years of experience, in-training) under the supervision of a board-certified radiologist (IrB, 10 years of experience). In case of disagreement, the case was discussed (InB, SB, IrB), the segmentation was deleted and the workflow was repeated. All investigators were blinded to the clinical data. The segmentation process was repeated by a radiologist (SM, 3 years of experience, in-training) to analyze the inter-observer variance.

#### Features extraction

Within the 3D Slicer software platform we used the open-source extension PyRadiomics to extract the radiomics features [10, 11]. We used the default settings of PyRadiomics and extracted all original standard features from seven feature classes: Shape-based, First Order Statistics, Gray Level Co-occurrence Matrix (GLCM), Gray Level Run Length Matrix (GLRLM), Gray Level Size Zone Matrix (GLSZM), Gray Level Dependence Matrix (GLDM), Neighboring Gray Tone Difference Matrix (NGTDM) leading to 107 features per segmentation as previously described [13].

### Evaluation of inter-observer reproducibility

Intra-class correlation coefficients (ICC) were calculated for each feature to assess the reproducibility of measurements [13] applying ICC3 of the Pingouin package [14] in Python. In short, ICC range from -1 (perfect anticorrelation) to 1 (perfect correlation) and reproducibility can be defined as excellent (≥ 0.75), good (0.60 – 0.74), moderate (0.40 – 0.59) or poor ≤ 0.39 [13].

### Quantitative radiographic biomarkers to predict overall survival

We stratified our patient cohort (I, complete cohort) into subgroups depending on the therapeutic approach (II, surgery [with and without radiation and/or chemotherapy] vs. III, definite radiochemotherapy vs. IV, definite radiotherapy) to assess the dedicated performance of radiographic biomarkers. All analysis were performed in Python 3.7.6, within Jupyter Notebook and respective open-source packages to ensure highest transparency and sustainability. Scikit-survival 0.16.1 was used for the survival analysis [15]. We applied and compared two variant machine learning models for automatic feature selection and survival prediction. In model 1, we standardized the feature values to account for scale differences among features and applied an elastic net (EN) with tuning of the penalty strength *alpha* applying grid-search. In model 2, we applied a Random Survival Forest (RSF) with permutation-based stratification of feature importance. In order to rule out inter-scanner variability, we limited the analysis to include only examinations from one single CT scanner and respectively, we had to analyze a rather small patient cohort and small patient subgroups. The stratification of patients in variant training and test datasets could highly impact the model performance. To account for this potentially confounding variability, we performed independent iterations with Monte Carlo cross-validation with 100 random splits for both models. We identified and ranked the absolute features’ values according to the mean and median of the 100 iterations to obtain the top 10 feature signature (either ranked by the mean or by the median). We depict ranking by the mean and median of the 100 iterations to point out the high impact of the averaging strategy on the feature selection. Each iteration of each model was trained on an independent dataset of 75% of the data drawn at random and tested in the remaining 25%. In summary, this approach accounts for performance differences of the models based on the different stratifications of the training and test datasets and homogenizes the results facing small patient subgroups. In the EN (model 1) additional nested fivefold shuffled cross-validation was used within the training-dataset for grid-search hyperparameter tuning. In model 1, the penalizing EN allowed for automatic feature selection and we ranked each feature’s median and mean of the 100 iterations to analyze the feature importance. In model 2, we ranked the feature’s median and mean of each of the 100 iterations according to the feature importance estimated by its permutation applying the ELI5 library (https://eli5.readthedocs.io/en/latest/overview.html). We illustrate the workflow of the model implementation in supplementary material S1. To analyze the potential complementary value of the signature features we performed correlation analyses. Of note, we use the term AUC to describe the Cox-Survival (Harrel’s) C (AUC).

#### General statistical analysis

Statistical analyses were performed in Python, using the Pingouin package [14]. Further statistics and graphical illustrations were performed in Microsoft Excel (Microsoft Corporation) and Affinity Designer 1.8.5.703 (Serif (Europe) Ltd). The sample size was the result of including all eligible patients since the installation of the used CT scanner in our department.

### Data availability

The datasets used and/or analyzed during the current study are available from the corresponding author on reasonable request. We calculated the radiomics quality score for our study and yielded a score of 16 (https://radiomics.world/rqs, supplementary material S2) [16].

## Results

### Study population

Our study population comprised 157 patients (male, 119; female, 38; age, 64.39 ± 10.71) who received baseline CT acquisition at a single CT scanner. Patients were treated with surgery (*n* = 47 with and without additional RT or RCTX), definite RCTX (*n* = 57) or definite RT (*n* = 20). In 16 patients, the treatment was unclear and 17 patients refused therapy. The latter two patient subgroups were excluded from dedicated subgroup analyses due to their small patient size. In total, 78.3% [123/157] of survival data records were censored. The proportion of censoring was pronounced after 2 years of follow-up (supplementary material S3). We depict the patient characteristics in Table 1 and Kaplan-Meier survival plots for each subgroup in supplementary material S4.

### Overall survival prognostication performance using elastic nets and random survival forests

**Fig. 3:**
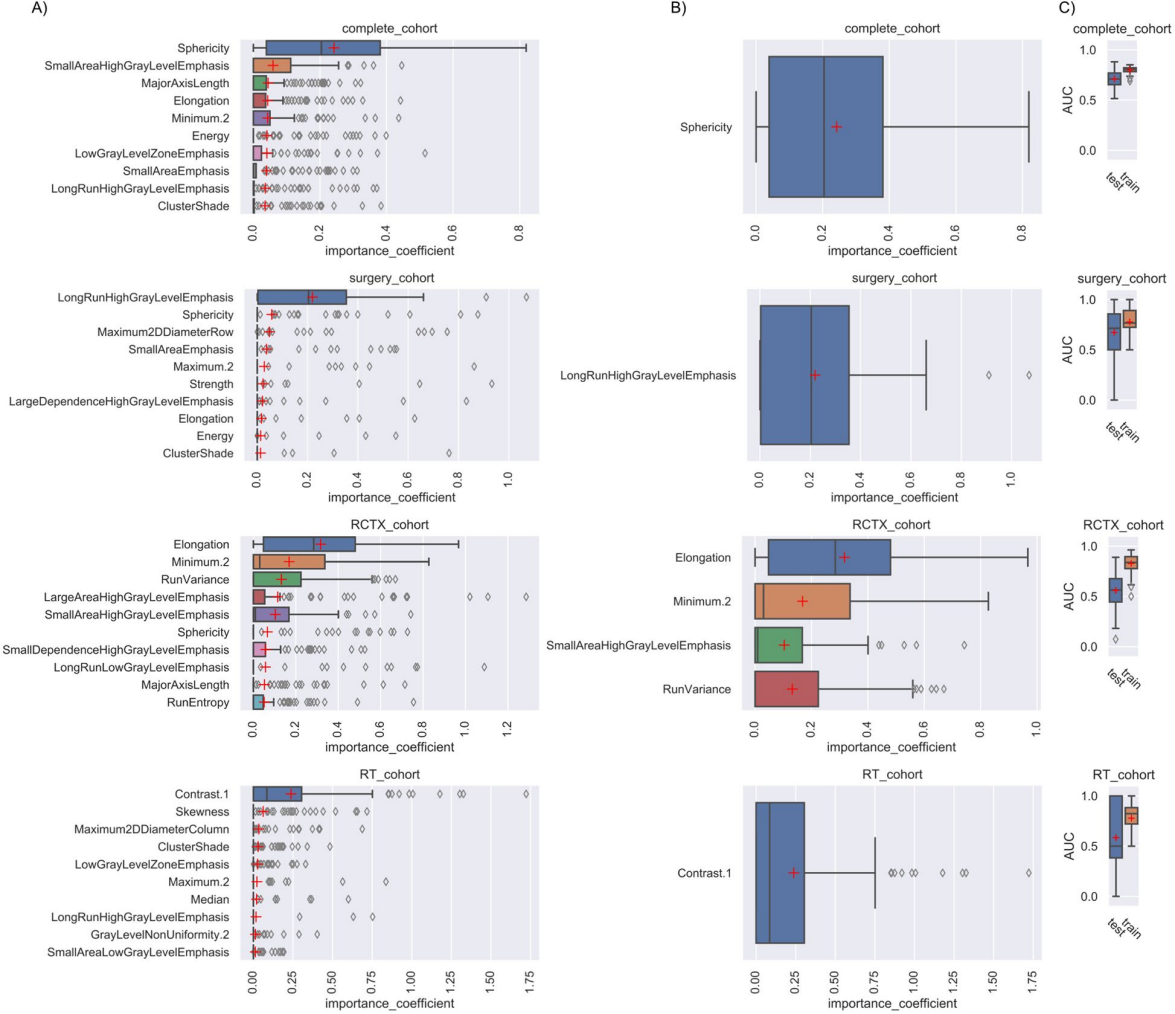
Top 10 elastic net features with importance ranking. Box-Whisker Plots depict the importance coefficient of each feature for each subgroup either ranked according to the mean (**A**) or median (**B**) of the Monte Carlo 100 random split cross-validation. In (**C**) the Cox-Survival (Harrel’s) C (AUC) is shown for each 100× iterated model. Only features with an importance coefficient >0 are shown

**Fig. 4:**
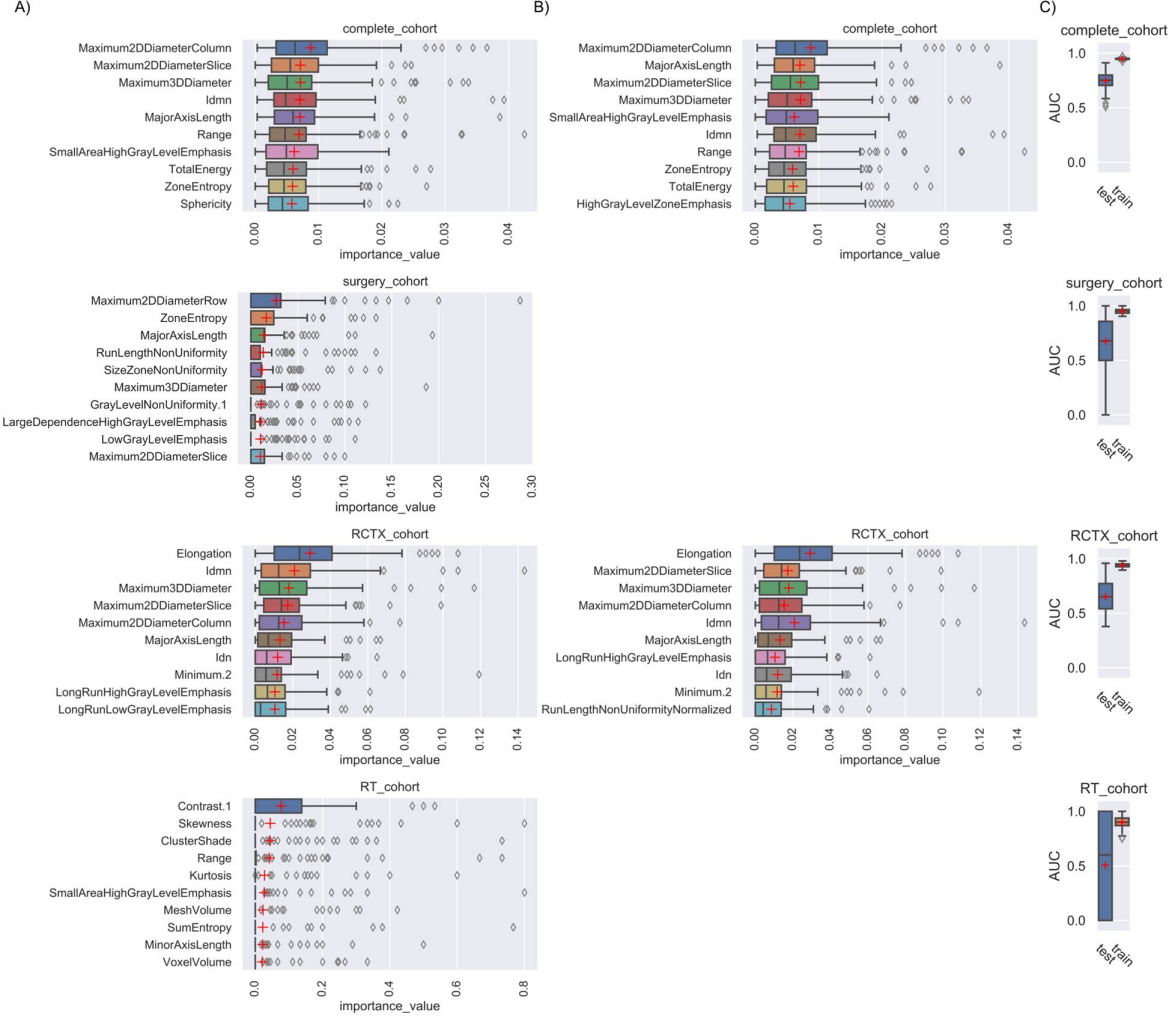
Top 10 random survival forest features with importance ranking. Box-Whisker Plots depict the importance value of each feature for each subgroup either ranked according to the mean (**A**) or median (**B**) of the Monte Carlo 100 random split cross-validation. In (**B**) the surgery and RT cohort did not yield any non-zero feature in the median ranked approach. In (**C**) the Cox-Survival (Harrel’s) C (AUC) is shown for each 100× iterated model. Only features with an importance value >0 are shown

The EN and RSF demonstrated variant prognostic performance depending on the analyzed subgroup (Table 2, Figs. 3 and 4). The complete cohort had moderate prognostic power (EN, AUC = 0.71 [95% CI 0.70–0.73]; RSF, AUC = 0.75 [95% CI 0.73–0.76]). The models showed moderate prognostic power for the surgery cohort (EN, AUC = 0.67 [95% CI 0.63–0.72]; RSF, AUC = 0.68 [95% CI 0.63–0.72]). The EN could not predict RCTX and RT treated patients’ survival accurately (AUC = 0.56–0.59). The RSF could weakly predict the survival of the RCTX cohort (AUC = 0.65 [95% CI 0.62–0.68]). No survival prognostication was seen for the RSF analyzing the RT cohort (AUC = 0.51 [95% CI 0.42–0.60]). The RSF prognostic performance was superior to the EN for the complete cohort (p = 0.002) and the RCTX cohort (*p* < 0.001).

**Table 2:** Model performance for the EN, RSF, benchmark and combined (quantitative imaging features and benchmark) models

Cohort	AUC test	95% CI	AUC train	95% CI	p(test) vs RSF	p(test) vs Benchmark or combined
EN						
Complete	0.711	0.695–0.728	0.799	0.793–0.805	0.002	< 0.001
Surgery	0.672	0.627–0.718	0.778	0.751–0.804	0.938	< 0.001
RCTX	0.560	0.527–0.593	0.827	0.808–0.846	< 0.001	0.420
RT	0.585	0.516–0.653	0.778	0.748–0.808	0.158	0.434
EN Benchmark						
Complete	0.632	0.613–0.650	0.745	0.730–0.760		
Surgery	0.445	0.404–0.486	0.772	0.741–0.804		
RCTX	0.578	0.546–0.611	0.789	0.771–0.808		
RT	0.621	0.560–0.681	0.776	0.751–0.801		
RSF						
Complete	0.746	0.731–0.760	0.948	0.946–0.949		< 0.001
Surgery	0.675	0.629–0.721	0.950	0.946–0.954		0.006
RCTX	0.652	0.622–0.681	0.938	0.935–0.942		0.002
RT	0.507	0.422–0.591	0.897	0.887–0.907		< 0.001
RSF Benchmark						
Complete	0.669	0.651–0.686	0.827	0.823–0.830		
Surgery	0.578	0.526–0.630	0.809	0.797–0.821		
RCTX	0.575	0.538–0.613	0.858	0.852–0.865		
RT	0.788	0.734–0.842	0.818	0.803–0.833		
EN combined						
Complete	0.799	0.793–0.805	0.798	0.792–0.805	< 0.001	0.065
Surgery	0.645	0.604–0.685	0.795	0.770–0.819	0.335	0.369
RCTX	0.555	0.525–0.586	0.842	0.820–0.864	< 0.001	0.853
RT	0.595	0.527–0.664	0.833	0.803–0.864	0.601	0.828
RSF combined						
Complete	0.759	0.745–0.772	0.949	0.948–0.951		0.215
Surgery	0.673	0.632–0.714	0.953	0.947–0.958		0.944
RCTX	0.650	0.615–0.684	0.943	0.939–0.956		0.924
RT	0.568	0.488–0.647	0.904	0.895–0.912		0.298

The Cox-Survival (Harrel's) C (AUC) with the respective confidence interval is shown for each model and subgroup for the training and test dataset. For statistical analysis two-sided, independent Student’s t-test was performed comparing the iterated AUC values of the test dataset

*AUC* Cox-Survival (Harrel’s) C, *EN* Elastic net, *RCTX* Radiochemotherapy, *RT* Radiotherapy, *RSF* Random survival forest

### Benchmarking against non-invasive clinical parameters

We benchmarked the quantitative imaging features based EN and RSF against EN and RSF models based on clinical information (sex, age, cTNM stage) (Table 2, supplementary materials S5 and S6). The clinical models achieved a performance of AUC = 0.45–0.63 for EN and of AUC = 0.58–0.79 for RSF. The quantitative EN model had a significantly better performance for all cohorts (*p* < 0.001) except the RCTX and RT cohort (p = 0.42; 0.43). The quantitative RSF model had a significantly better performance for all cohorts (*p* ≤ 0.006) except the RT cohort. A combined model comprising the quantitative imaging features and clinical features did not improve the performance of the quantitative imaging models (*p* ≥ 0.07 for all features, Table 2, supplementary materials S7 and S8).

### Feature importance using elastic nets and random survival forests

**Fig. 5:**
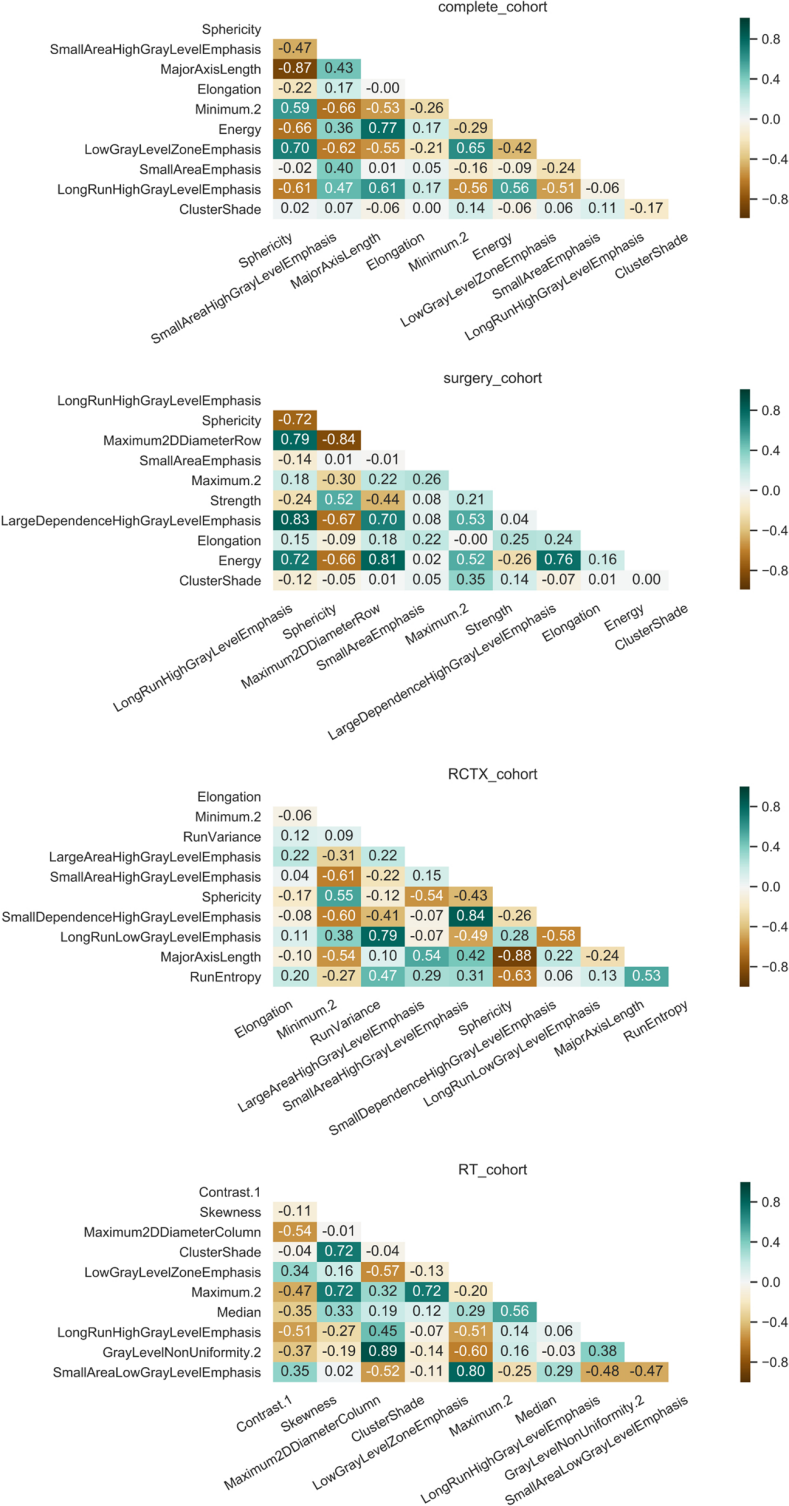
Correlation matrix of the top ranked features of the elastic net. The correlation matrices of the top features ranked by mean of Monte Carlo 100 random split cross-validation with elastic net are shown for each subgroup

**Fig. 6:**
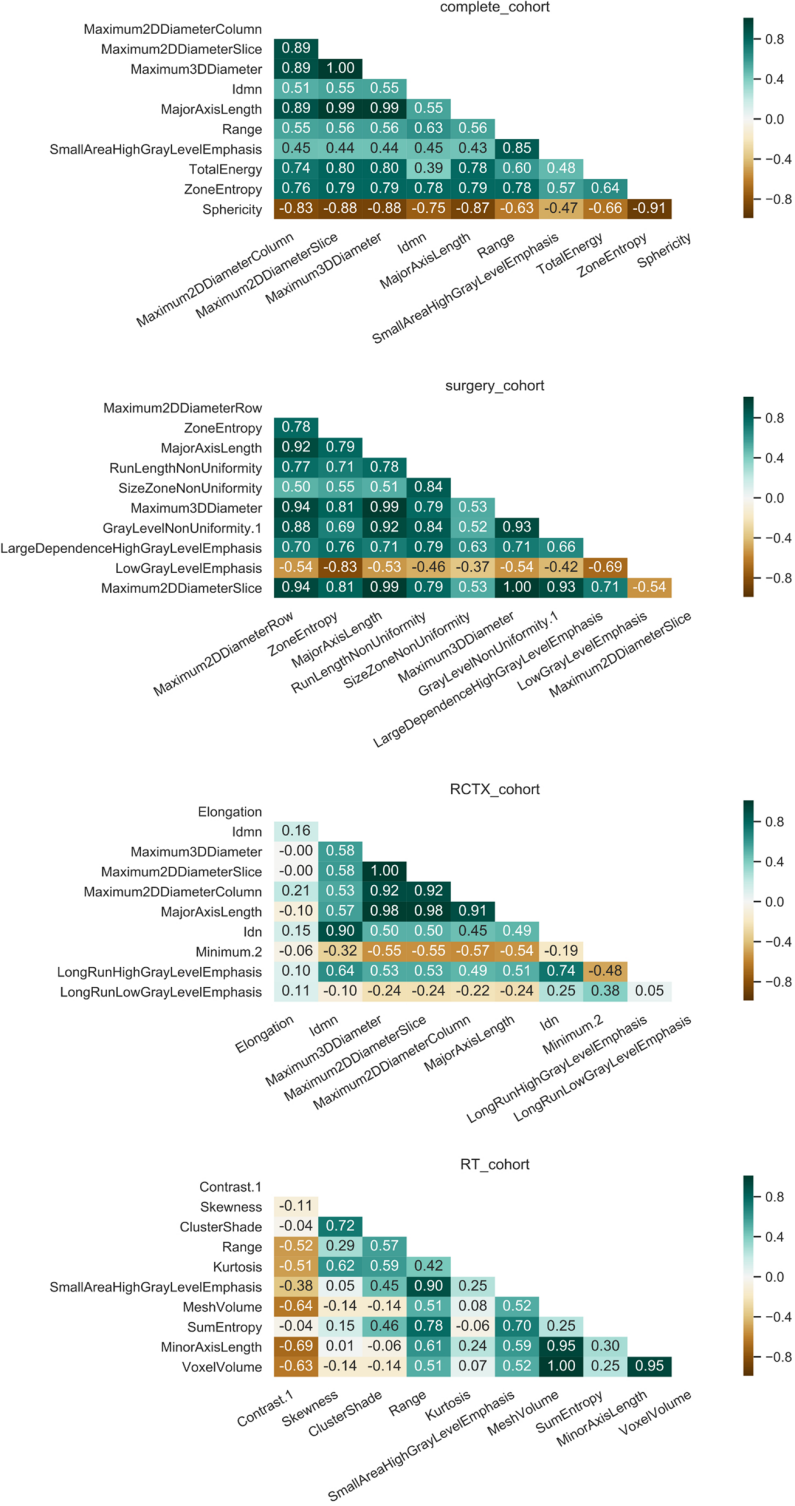
Correlation matrix of the top ranked features of the random survival forest. The correlation matrices of the top features ranked by mean of Monte Carlo 100 random split cross-validation with random survival forest are shown for each subgroup

As we assumed that the radiographic differences which influence the overall survival differ according to the therapeutic strategy and machine learning model, we ranked and compared the features’ importance values to depict the top 10 non-zero features. The EN tended to favor one dedicated feature for each subgroup, especially when using the median as averaging strategy. This highlights the high variation of feature importance depending on the different stratification of patients in the iterated train/ test splits (if a feature has a median of zero it means that the feature did not have any predictive importance in more than half (> 50) of the model’s iterations). In EN (Fig. 3) the top ranked feature was a shape feature for the complete cohort (Sphericity) and RCTX (Elongation). In the surgery cohort, the top ranked EN feature was part of the gray level run length matrix features (LongRunHighGrayLevelEmphasis). The RSF revealed larger sets of features with an importance coefficient >0 and more subtle differences between the ranked feature importance (Fig. 4). In RSF the top ranked feature was a shape feature for the complete cohort (Maximum2DDiameterColumn), surgery cohort (Maximum2DDiameterRow) and RCTX (Elongation). In the RT cohort the best ranked feature was Contrast (GLCM). Of note, the surgery and RT cohort did not yield any non-zero feature in the median ranked approach. To analyze the complementary information of lower ranked features we computed correlation analyzes of the top 10 mean ranked features for EN and RSF (Figs. 5 and 6). In both models a multitude of features are not strongly correlated (Figs. 5 and 6) depicting their potentially contributing value in the individual iterations.

In the clinical benchmark importance analysis, cT4, cT3 and cN3 yielded the highest EN importance and cT4 and patient age yielded the highest importance in the RSF model (supplementary materials S5 and S6). In the combined features (clinical and quantitative imaging) importance analysis, clinical features were found among the top ranked features, especially in EN (supplementary materials S7 and S8). The clinical benchmark characteristics revealed minimal correlation (supplementary materials S9 and S10). The combined model’s characteristics revealed weak correlations (supplementary materials S11 and S12).

### Inter-observer variance

Radiomics feature robustness was assessed by intra-correlation analysis and yielded excellent mean results for all feature classes (supplementary material S13 depicts the box whisker plots and detailed ICC values). Mean feature class ICC values ranged from 0.77 (std 0.19, GLSZM) to 0.96 (std 0.04, shape). Two radiomic features had ICC values <0.4 (poor, LargeAreaHighGrayLevelEmphasis, LargeDependenceHighGrayLevelEmphasis) (supplementary material S14). The EN never ranked poor features in the median averaging approach among the top 10 non-zero features. The EN and RSF never ranked features with poor ICC among the top 3 most important features. Poor ICC features were among the top 10 ranked features in the surgery (rank 7) and RCTX (rank 4) cohort of the EN and the surgery cohort (rank 8) of the RSF.

## Discussion

Artificial intelligence (AI) is able to integrate and synthesize high-dimensional data [4]. Narrow-task AI applications need to be interpretable to bridge the translational gap [4]. Squamous cell carcinoma’s of the head and neck potentially multi-modal treatment is complex bearing optional treatment steps and the risk of long-term toxicities [3]. AI might yield the potential to aid clinical decision making at best to improve patient outcome. Our data demonstrate that the quantitative image analysis of standard-of-care baseline CT examinations has the potential to predict the overall survival of head and neck carcinoma patients. We were able to demonstrate that a random survival forest was superior to an elastic net for overall survival prognostication. The random survival forest-model, trained on quantitative image data was superior to the respective clinical benchmark. We ranked the image features according to their importance for each model in order to improve the interpretability of the models. In penalizing models, ranking of feature’s importance according to the iterated median seems to improve the interpretability of the model to a higher degree than following a mean-ranking approach. Shape features had the highest prognostic impact followed by higher dimensional radiomics features.

Radiomics’ prognostic potential in head and neck cancer patients was shown in numerous studies [9, 17–19]. Welch et al. [17] demonstrated that signature radiomics features might be surrogates of tumor volume and they urged refinement of radiomic methodology by proposing a set of safeguards to promote sustainable radiomic research. In our work we followed the proposed safeguards [17]. We benchmarked our models against clinical factors [17] to demonstrate potential superiority of imaging biomarkers. We used a penalizing elastic net and a random survival forest ensemble method with ranking of features according to their model-importance to select the highest ranked imaging features with subsequent analysis of multicollinearity and underlying dependencies [17]. The ranking of the features was done after 100 random iterations and we either used the mean or median of the features’ importance value. The averaging strategy had a high impact on the feature’s rank. In our cohort, most features that were low ranked applying the mean as averaging method did not yield any importance in more than 50% of the iterations using an EN (as depicted by a median = 0). Thus, one might propose using the median as averaging strategy as it seems to depict a clearer image of the high degree of variation in feature importance in different train/test splits. This might be a result of the cohort and subgroup sizes but also a feature intrinsic effect and we urge caution in interpreting studies with similar size if only one train/test split was performed. Nevertheless, the median averaging approach might disguise whole feature sets of similar importance, e.g., if three features perform equally well each random split might pick one feature randomly and each feature could obtain an importance value >0 in less than 50% of the iterations. Consequently, one would dismiss the whole feature set in the median approach. That contrast (GLCM) was the top ranked feature in the RT cohort was potentially the result of bias facing a small patient subgroup size with approx. 50% right censoring. We did not pre-exclude features with poor ICC in order to analyze the capability of our models to automatically exclude non-robust features. In three models (EN, 2; RSF, 1) features with poor ICC were ranked among the top 10 most important features, but never among the top 3, indicating that most of the models worked well in automatically excluding potentially non-robust features. The prognostic performance of our results (AUC = 0.71–0.75 for our best working models) are in line with prior studies, i.e. Aerts et al. [9] had a performance as measured by the Concordance Index (CI) as generalization of the AUC from 0.69 and Patel et al. [19] revealed performances of 0.79 in a combined clinical and radiomics model. In line with Welch et al. [17] the majority of our models ranked shape features with highest feature importance. Contrary to Welch et al. [17] and in line with the radiomics hypothesis [7] and further studies [9, 19, 20] our results indicate that radiomics features beyond shape features inherit complementary value which might be necessary to build high performing machine learning models. We provided novelty by following the recently proposed pathway of clinical AI translation in designing narrow-task AI applications [4] – we designed each model for each therapeutic subgroup. Our results indicate that variant treatment subgroups’ prognostication depends on variant radiomics features. This finding does not only promote the interpretability of the models to path the way of bridging the translational gap but also shows the potential of radiomics to aid clinical decision making. We hypothesize that radiomics imaging biomarker could aid in the stratification of patients into respective treatment strategies i.e. if a patient might present with imaging traits that are associated with diminished survival in one but prolonged or unaffected survival in another treatment-specific survival-model. Large prospective multicenter studies are necessary to stratify generalizable feature candidates for aiding treatment selection and to path the way of integrating radiomics in clinical tumor board meetings. Our study has limitations that warrant discussion. First, the retrospective nature of our study might inherit selection bias. To obtain a reasonably sized study cohort for machine learning development, we included SCCHNs with variant localizations and HPV-status though known variation in outcome [2, 3, 21]. To analyze treatment-specific imaging biomarkers we stratified subgroups which tended to be small sized and hence the generalizability of the subgroup results should not be overstated. Right-censored data is a common problem in survival analysis and availability of more uncensored survival data would have been favorable. Last, to rule out inter-scanner variability we had to perform a single center approach, though this approach might reduce generalizability of our results.

## Conclusions

In conclusion, this work demonstrates that standard of care baseline CT imaging of SCCHN patients can be used for the computational extraction of imaging biomarkers that allow treatment specific outcome prognostication. These biomarkers may provide objective response estimates as additional tool to facilitate and improve individualized tumor board consensus. Imaging biomarkers were superior to clinical features in outcome prediction. Treatment specific imaging biomarker importance ranking might yield the potential to serve as a tool in clinical practice in aiding stratification of patients into appropriate treatment arms to improve outcomes.

## Supplementary Information

**Additional file 1: S1.** Workflow of model development. **S2.** Radiomics quality score. **S3.** Supplementary Figure 1. **S4.** Supplementary Figure 2. **S5.** Supplementary Figure 3. **S6.** Supplementary Figure 4. **S7.** Supplementary Figure 5. **S8.** Supplementary Figure 6. **S9.** Supplementary Figure 7. **S10.** Supplementary Figure 8. **S11.** Supplementary Figure 9. **S12.** Supplementary Figure 10. **S13.** Intraclass correlation analysis: radiomics feature classes. **S14.** Intraclass correlation analysis: individual radiomics features.

## Data Availability

The datasets used and/or analysed during the current study are available from the corresponding author on reasonable request.

## Abbreviations

AI: Artificial intelligence
AUC: Cox-Survival (Harrel’s) C (AUC)
EN: Elastic net
ICC: Inter-class correlation coefficient
RSF: Random survival forest
SCCHN: Squamous cell carcinoma of the head and neck
GLCM: Gray Level Co-occurrence Matrix
GLRLM: Gray Level Run Length Matrix
GLSZM: Gray Level Size Zone Matrix
GLDM: Gray Level Dependence Matrix
NGTDM: Neighboring Gray Tone Difference Matrix
