# Supplementary material for: Radiomics for therapy-specific head and neck squamous cell carcinoma survival prognostication (part I)
Source: BMC Med Imaging. 2023 Jun 2;23:71. doi: 10.1186/s12880-023-01034-1 (PMC10236822; doi:10.1186/s12880-023-01034-1)
Supplement: Supplementary file 1 — Additional file 1: S1. Workflow of model development. S2. Radiomics quality score. S3. Supplementary Figure 1. S4. Supplementary Figure 2. S5. Supplementary Figure 3. S6. Supplementary Figure 4. S7. Supplementary Figure 5. S8. Supplementary Figure 6. S9. Supplementary Figure 7. S10. Supplementary Figure 8. S11. Supplementary Figure 9. S12. Supplementary Figure 10. S13. Intraclass correlation analysis: radiomics feature classes. S14. Intraclass correlation analysis: individual radiomics features. [file 12880_2023_1034_MOESM1_ESM.docx]

**Radiomics for therapy-specific head and neck squamous cell carcinoma survival prognostication (part I)**

***Supplementary Material***

Simon Bernatz^1^^,2,3^, MD; Ines Böth^1^, Ms; Jörg Ackermann^4^, PhD; Iris Burck^1^, MD; Scherwin Mahmoudi^1^, MD; Lukas Lenga^1^, MD; Simon S. Martin^1^, MD; Jan-Erik Scholtz^1^, MD; Vitali Koch, MD^1^; Leon D. Grünewald^1^, MD; Ina Koch^4^, PhD; Timo Stöver^5^, MD; Peter J. Wild^2,6^, MD; Ria Winkelmann^2^, MD; Thomas J. Vogl^1^, MD; Daniel Pinto dos Santos^1,7^, MD

^1^Department of Diagnostic and Interventional Radiology, University Hospital Frankfurt, Goethe University Frankfurt am Main, Theodor-Stern-Kai 7, 60590 Frankfurt am Main, Germany;

^2^Dr. Senckenberg Institute for Pathology, University Hospital Frankfurt, Goethe University Frankfurt am Main, 60590, Frankfurt am Main, Germany;

^3^Frankfurt Cancer Institute (FCI), 60590, Frankfurt am Main, Germany;

^4^Department of Molecular Bioinformatics, Institute of Computer Science, Johann Wolfgang Goethe-University, Robert-Mayer-Str. 11-15, 60325 Frankfurt am Main, Germany;

^5^Department of Otorhinolaryngology, University Hospital Frankfurt, Goethe University Frankfurt am Main, Theodor-Stern-Kai 7, 60590 Frankfurt am Main, Germany;

^6^Frankfurt Institute for Advanced Studies (FIAS), 60438, Frankfurt am Main, Germany;

^7^Department of Diagnostic and Interventional Radiology, University of Cologne, Faculty of Medicine and University Hospital Cologne, Kerpener Str. 62, 50937, Cologne, Germany.

[**S1. Workflow of model development** 4](#_Toc93048366)

[**S2. Radiomics quality score** 5](#_Toc93048367)

[**S3. Supplementary Figure 1** 8](#_Toc93048368)

[**S4. Supplementary Figure 2** 9](#_Toc93048369)

[**S5. Supplementary Figure 3** 10](#_Toc93048370)

[**S6. Supplementary Figure 4** 11](#_Toc93048371)

[**S7. Supplementary Figure 5** 12](#_Toc93048372)

[**S8. Supplementary Figure 6** 13](#_Toc93048373)

[**S9. Supplementary Figure 7** 14](#_Toc93048374)

[**S10. Supplementary Figure 8** 15](#_Toc93048375)

[**S11. Supplementary Figure 9** 16](#_Toc93048376)

[**S12. Supplementary Figure 10** 17](#_Toc93048377)

[**S13. Intraclass correlation analysis: radiomics feature classes** 18](#_Toc93048378)

[**S14. Intraclass correlation analysis: individual radiomics features** 19](#_Toc93048379)

# **S1. Workflow of model development**


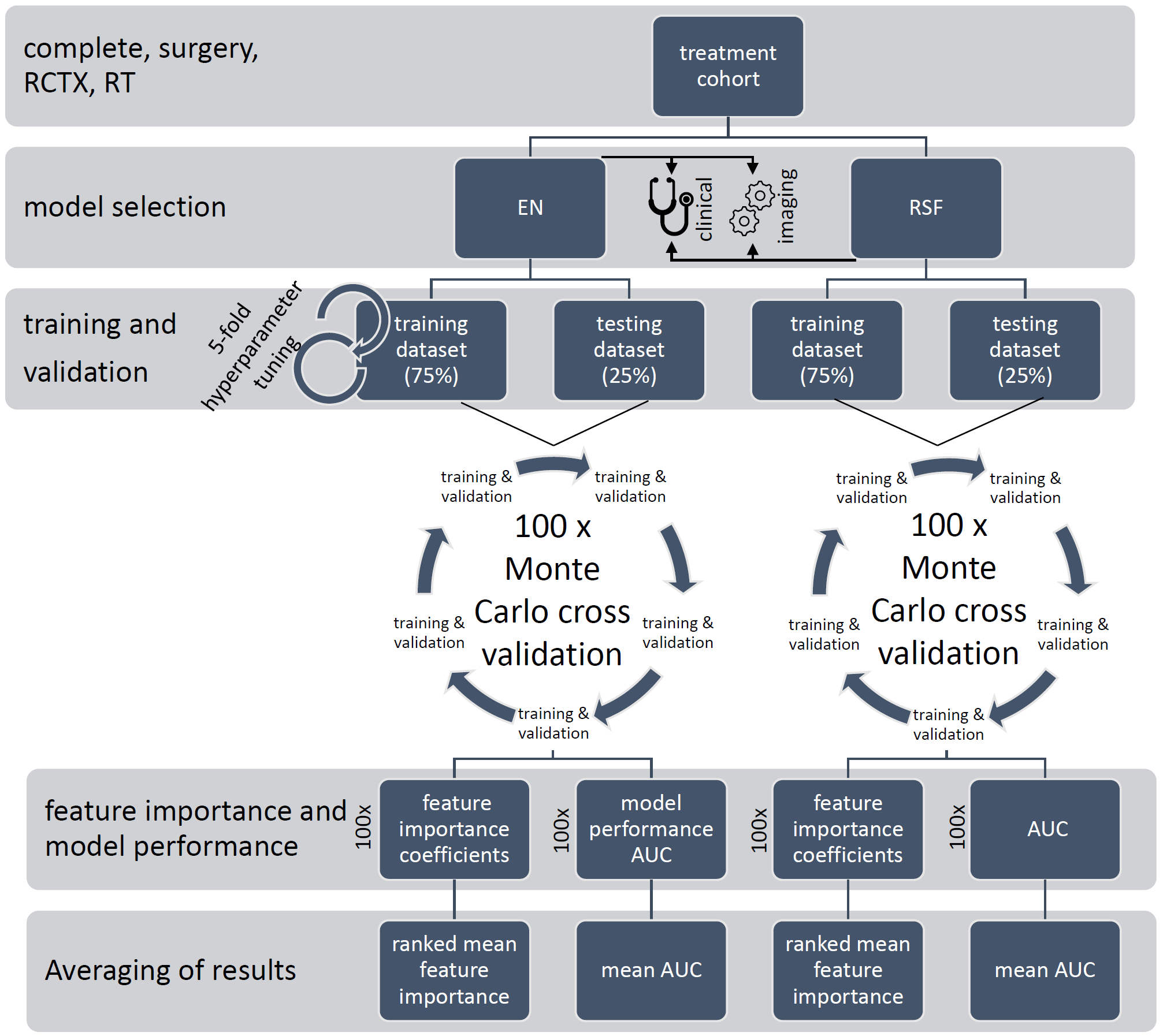


AUC, Cox-Survival (Harrel’s) C (AUC); EN, elastic net RCTX, radiochemotherapy; RSF, random survival forest; RT, radiotherapy

# **S2. Radiomics quality score**

# **S3. Supplementary Figure 1**


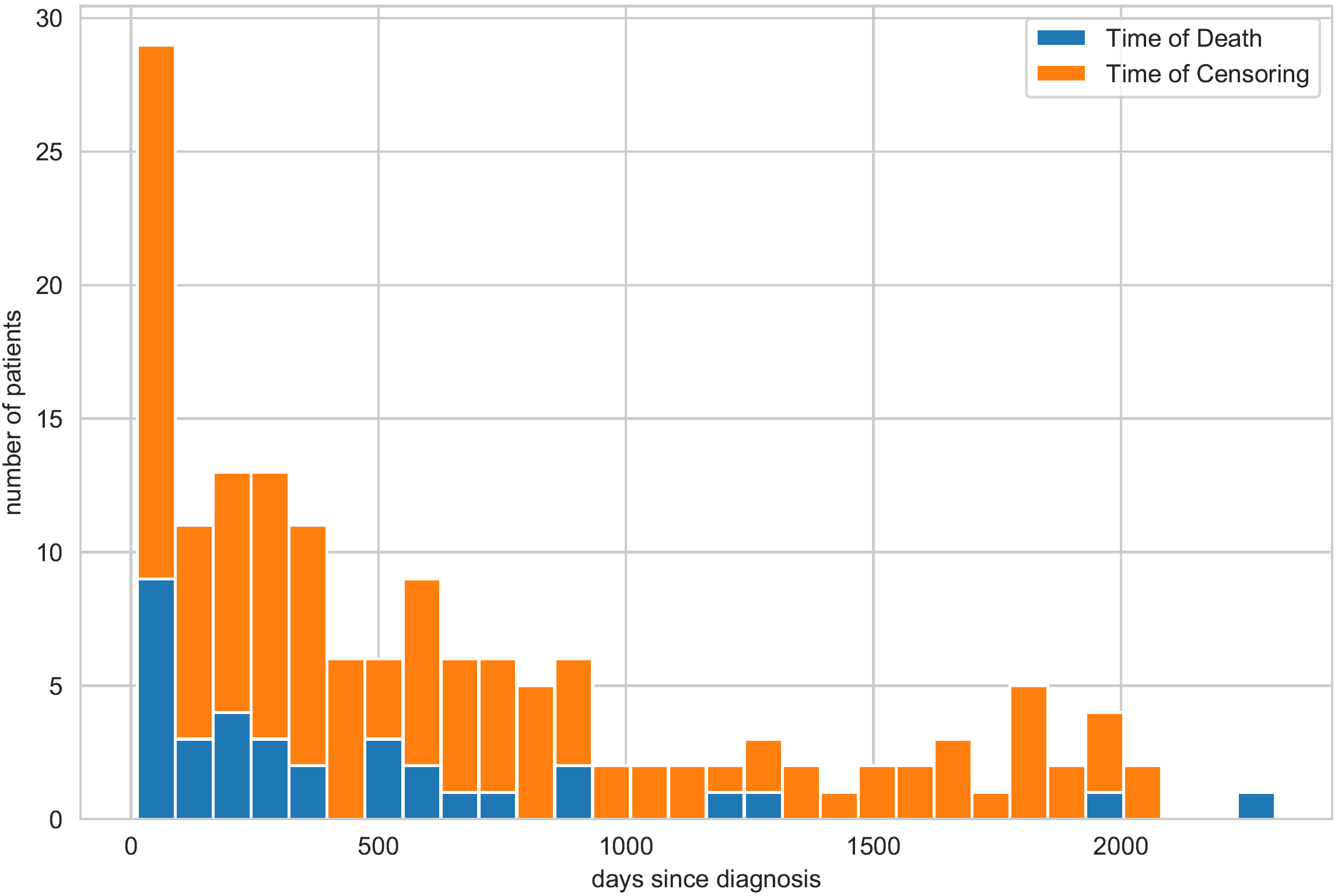


**Supplementary Figure 1.** *Barplot to visualize the time of death and censoring of the complete study cohort.*

# **S4. Supplementary Figure 2**

**
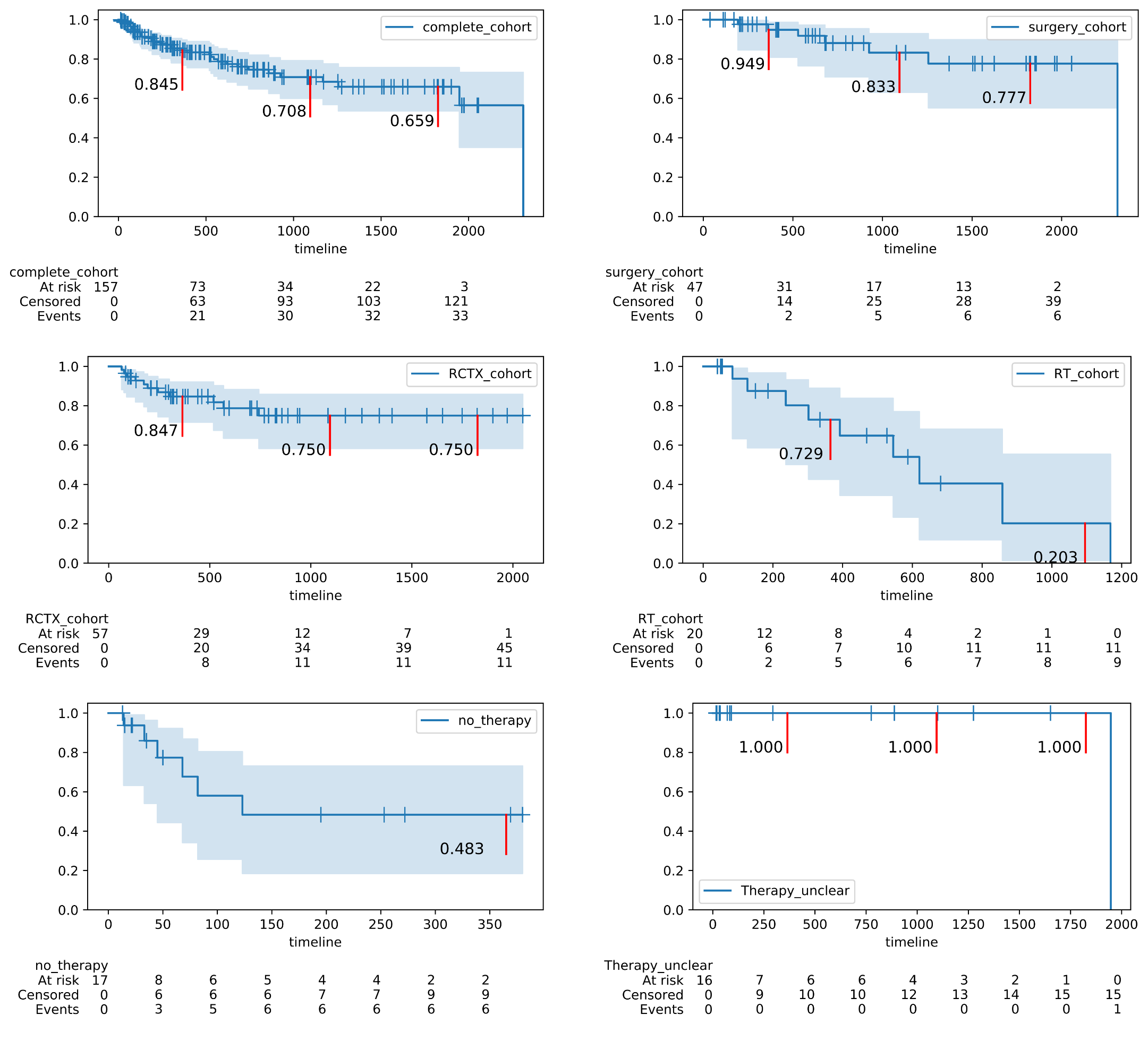
**

**Supplementary Figure 2.** *Survival estimates for each patient subgroup*

Kaplan-Meier survival plots with red lines depicting the proportion of estimated living patients for the timepoints of 1 year, 3 years and 5 years.

# **S5. Supplementary Figure 3**


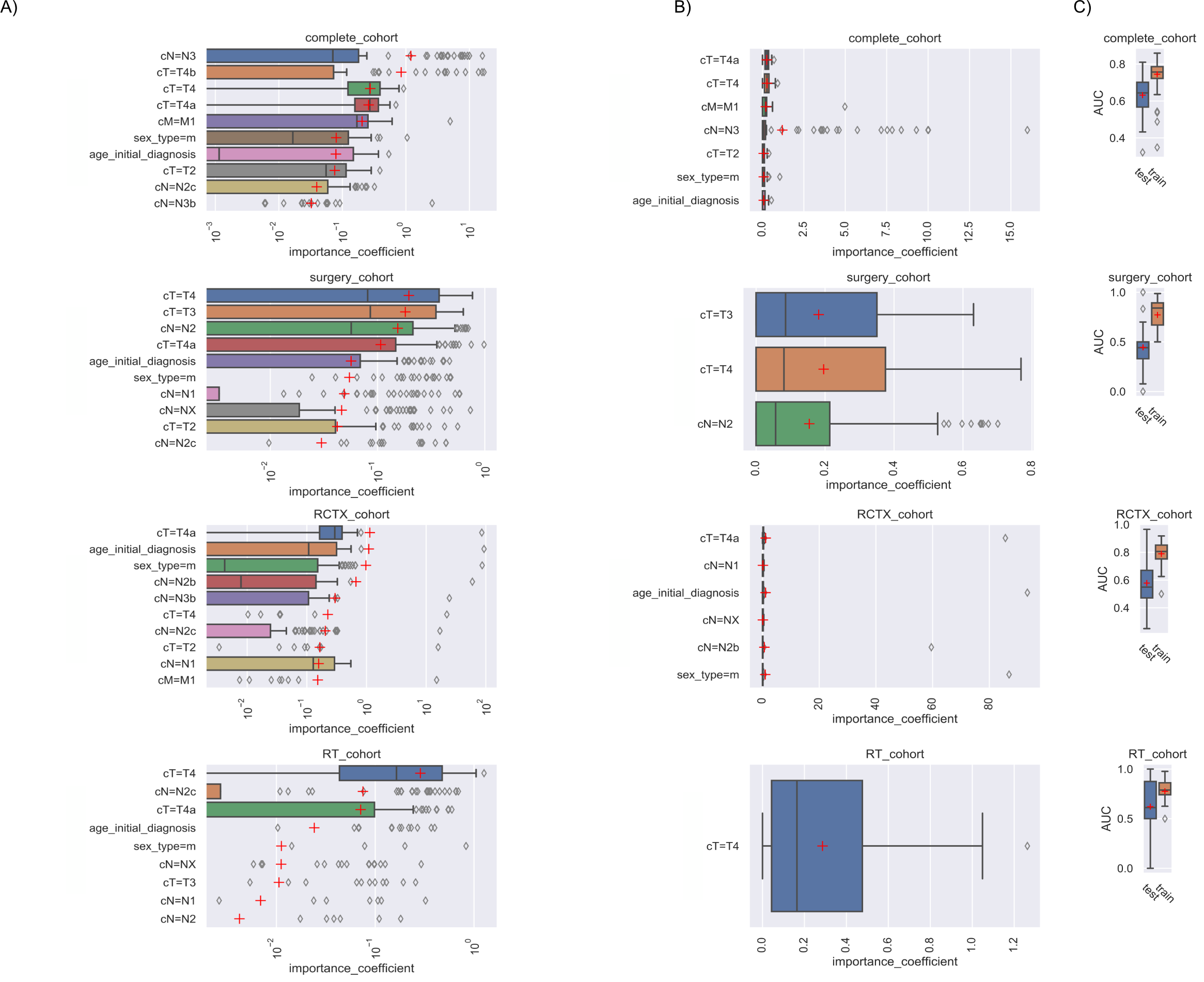


**Supplementary Figure 3.** *Top 10 elastic net clinical benchmark features with importance ranking*

Box-Whisker Plots depict the importance coefficient of each feature for each subgroup either ranked according to the mean (A) or median (B) of the Monte Carlo 100 random split cross-validation. In C) the Cox-Survival (Harrel’s) C (AUC) is shown for each final model. Only features with an importance coefficient > 0 are shown.

# **S6. Supplementary Figure 4**


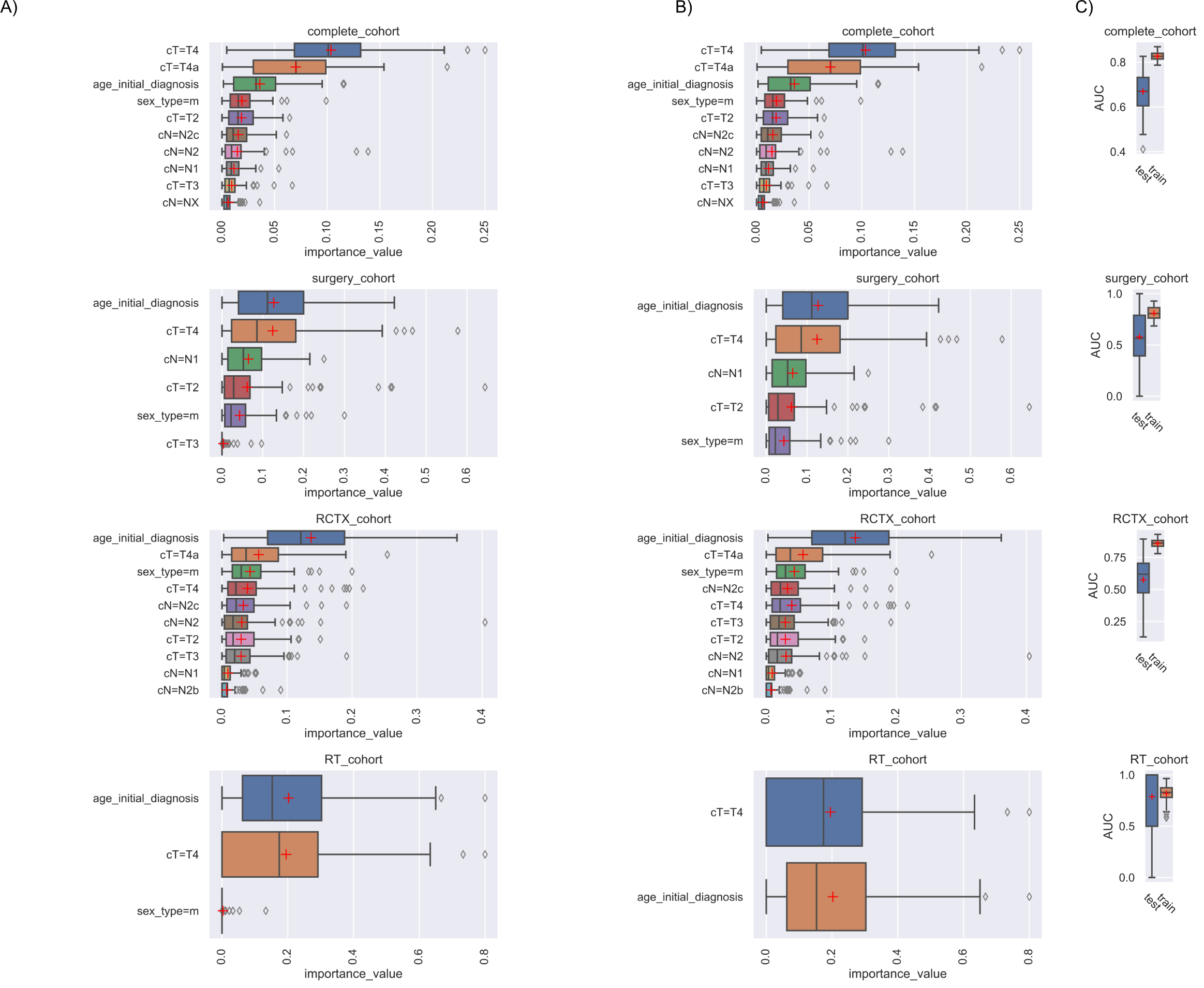


**Supplementary Figure 4.** *Top 10 random survival forest clinical benchmark features with importance ranking*

Box-Whisker Plots depict the importance value of each feature for each subgroup either ranked according to the mean (A) or median (B) of the Monte Carlo 100 random split cross-validation. In C) the Cox-Survival (Harrel’s) C (AUC) is shown for each final model. Only features with an importance value > 0 are shown.

# **S7. Supplementary Figure 5**


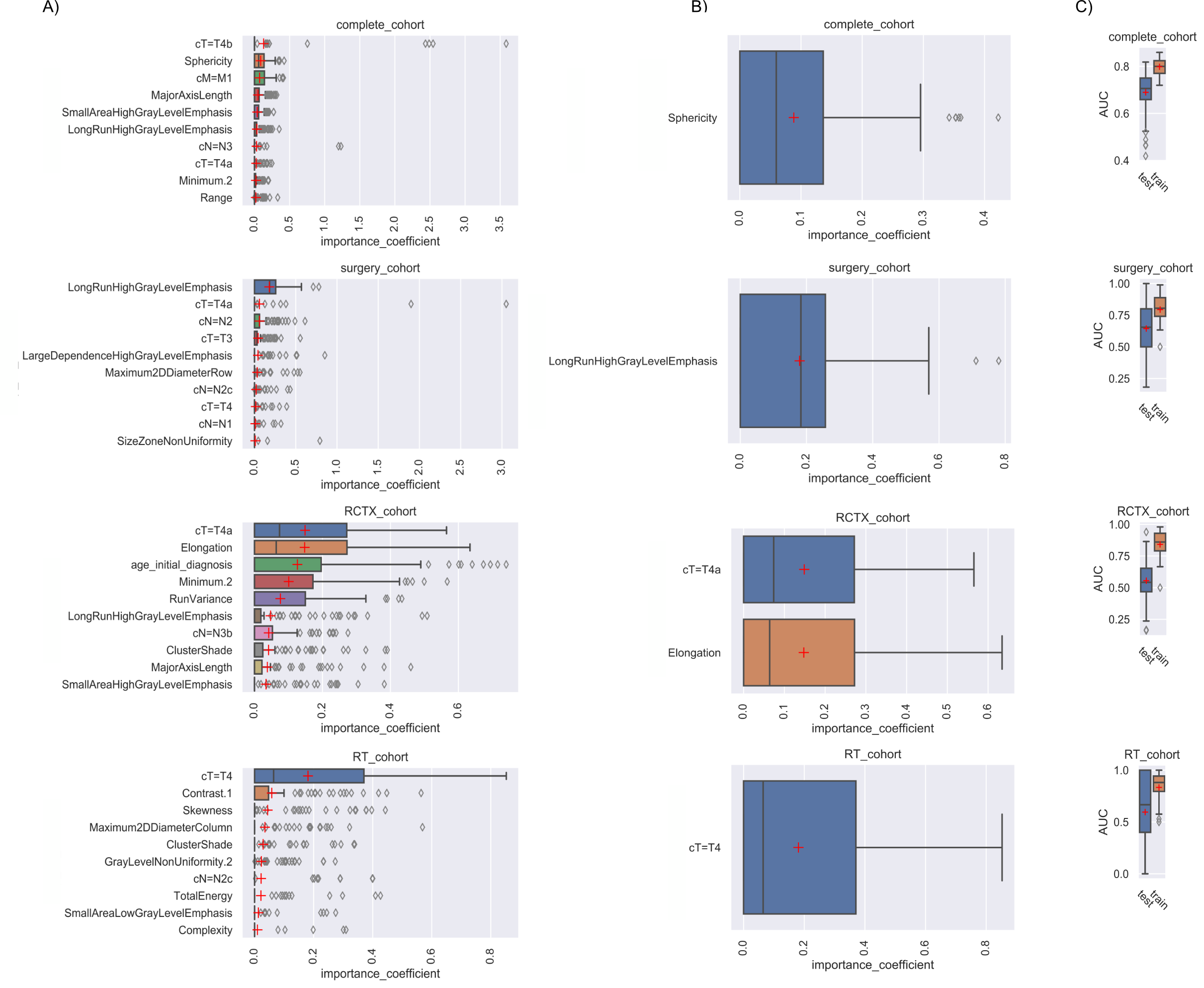


**Supplementary Figure 5.** *Top 10 elastic net combined features (clinical features and quantitative imaging features) with importance ranking*

Box-Whisker Plots depict the importance coefficient of each feature for each subgroup either ranked according to the mean (A) or median (B) of the Monte Carlo 100 random split cross-validation. In C) the Cox-Survival (Harrel’s) C (AUC) is shown for each final model. Only features with an importance coefficient > 0 are shown.

# **S8. Supplementary Figure 6**


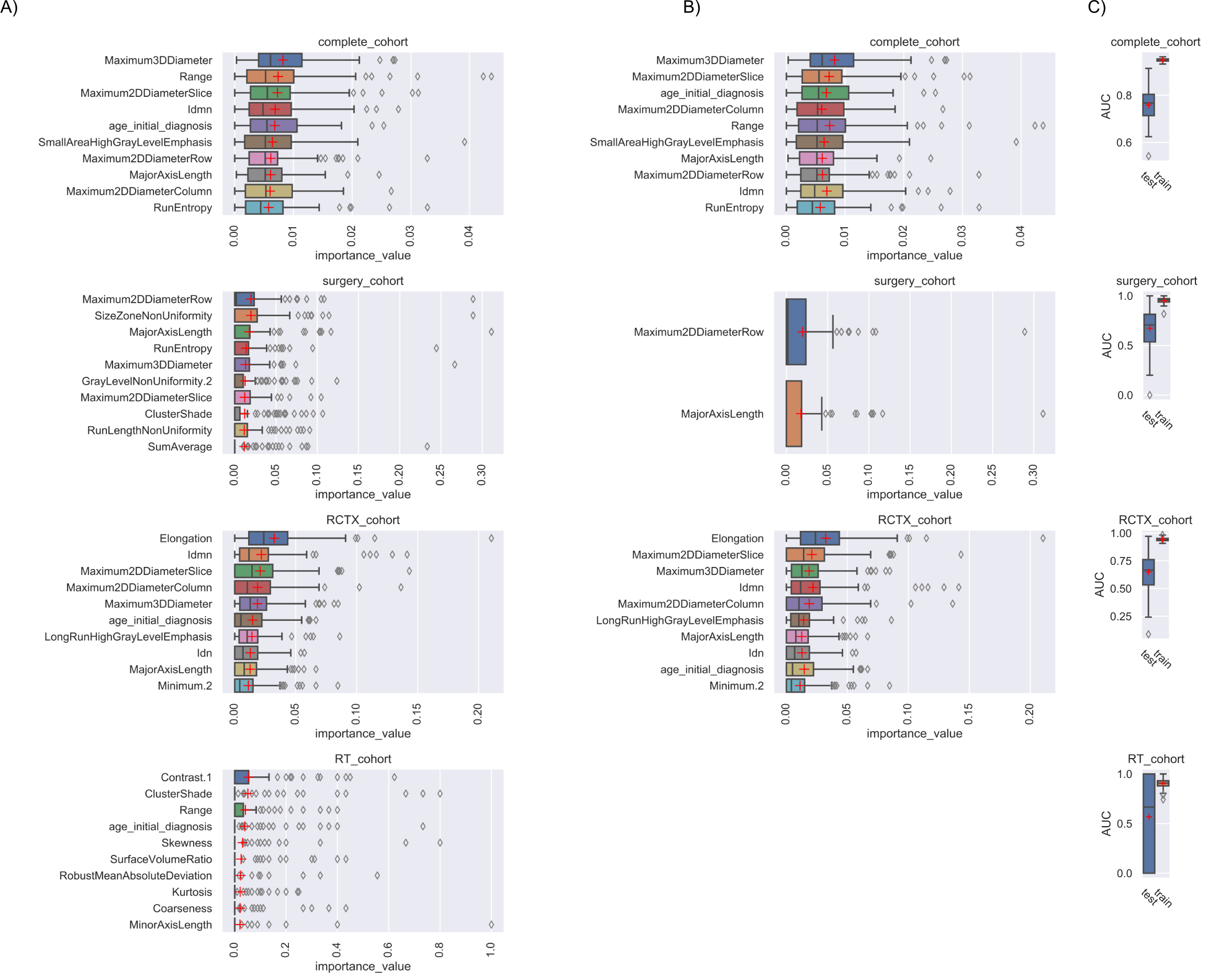


**Supplementary Figure 6.** *Top 10 random survival forest combined features (clinical features and quantitative imaging features) with importance ranking*

Box-Whisker Plots depict the importance value of each feature for each subgroup either ranked according to the mean (A) or median (B) of the Monte Carlo 100 random split cross-validation. In B) the RT cohort did not yield any non-zero feature in the median ranked approach. In C) the Cox-Survival (Harrel’s) C (AUC) is shown for each final model. Only features with an importance value > 0 are shown.

# **S9. Supplementary Figure 7**

**
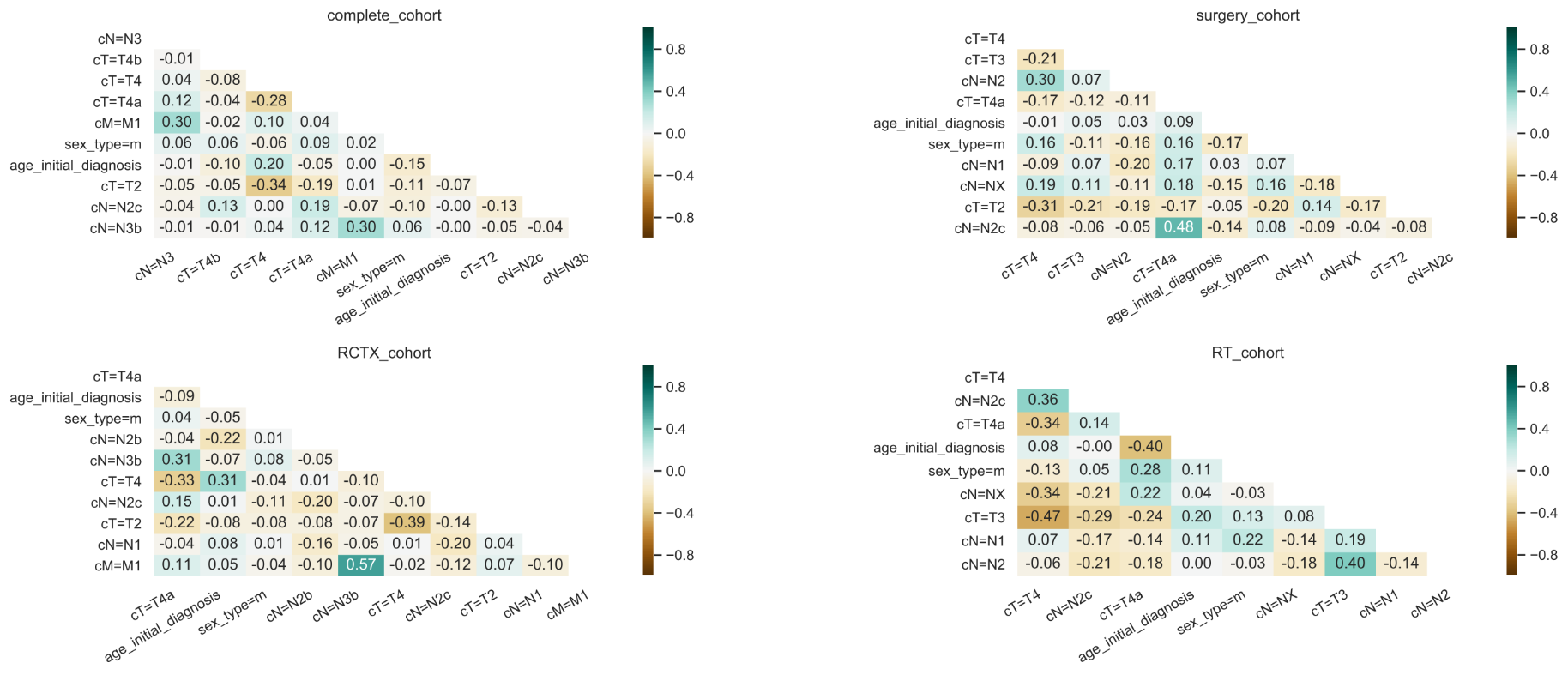
**

**Supplementary Figure 7:** *Correlation matrix of the top ranked clinical benchmark features of the elastic net*

The correlation matrices of the top ranked clinical benchmark features of the elastic net are shown for each subgroup.

# **S10. Supplementary Figure 8**

**
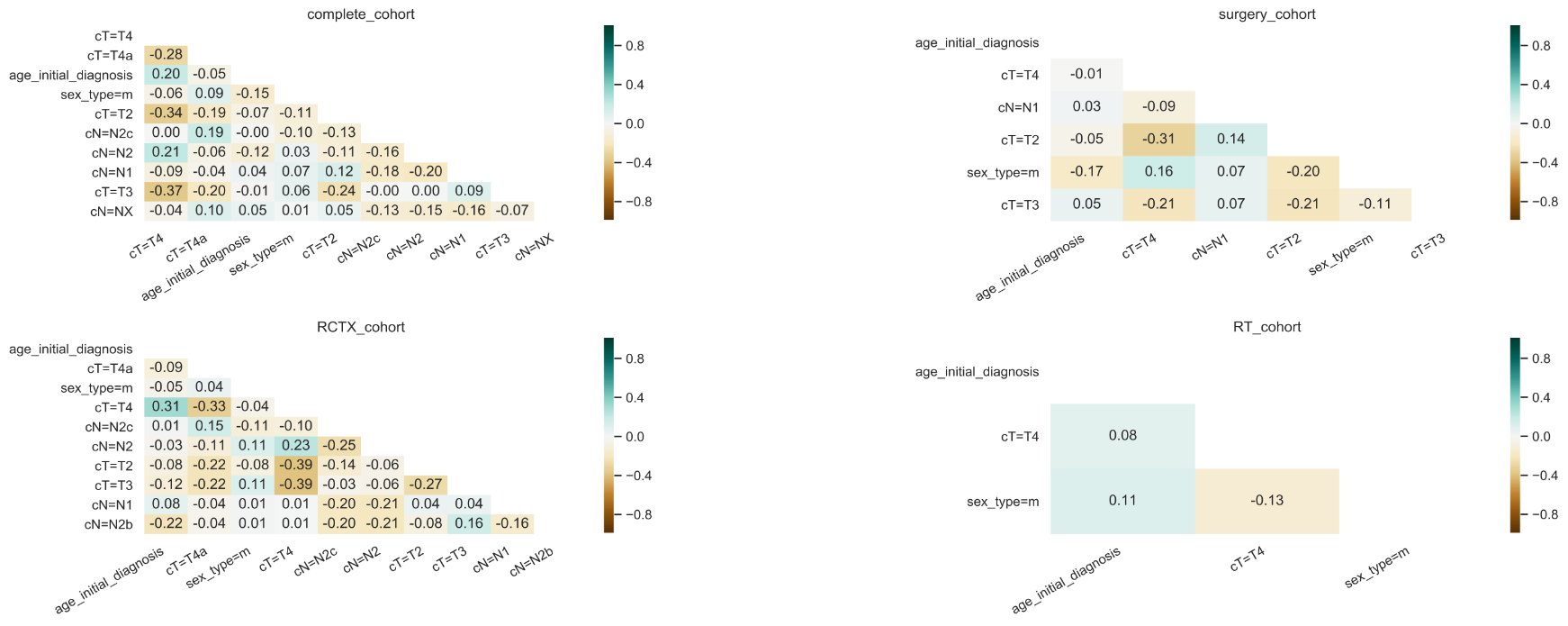
**

**Supplementary Figure 8:** *Correlation matrix of the top ranked clinical benchmark features of the random survival forest*

The correlation matrices of the top ranked clinical benchmark features of the random survival forest are shown for each subgroup.

# **S11. Supplementary Figure 9**


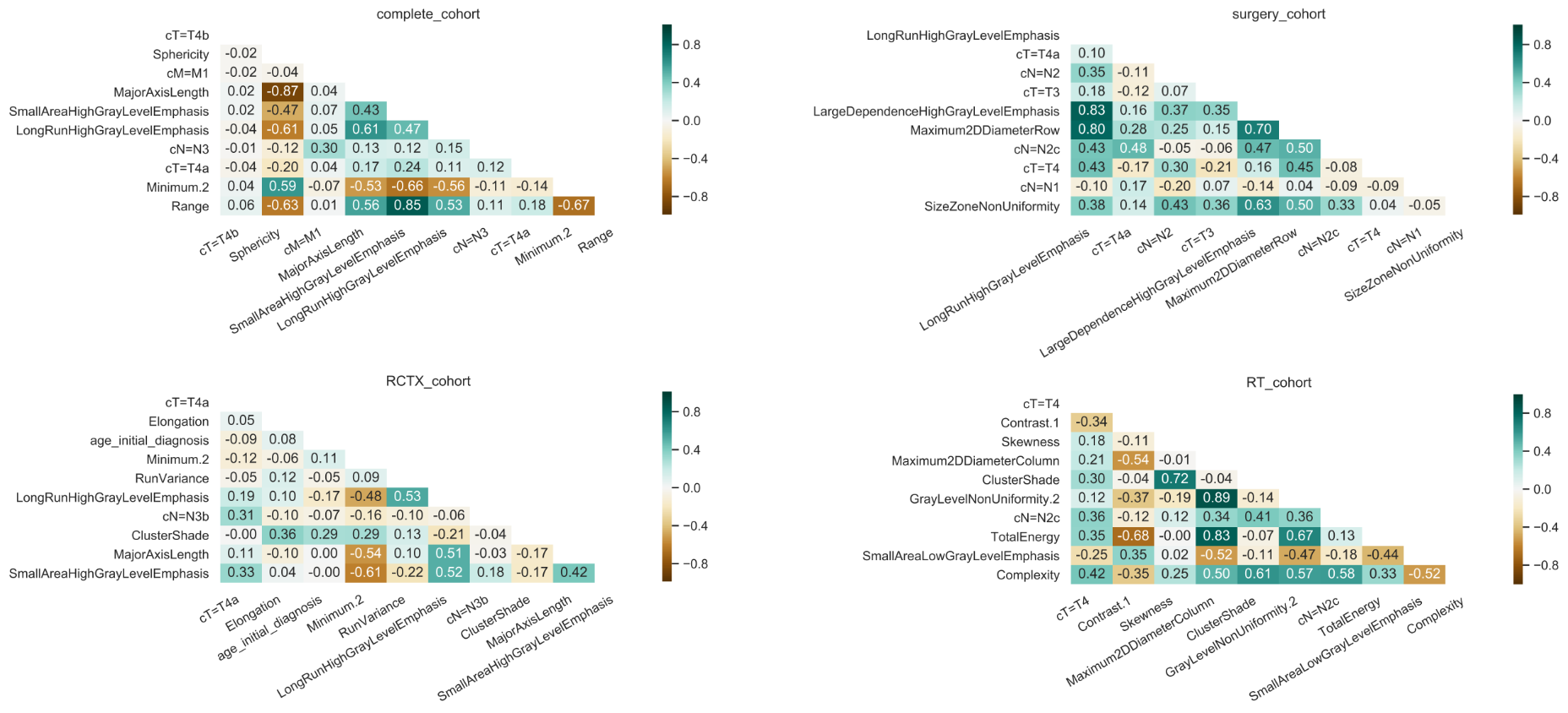


**Supplementary Figure 9:** *Correlation matrix of the top ranked combined features (clinical features and quantitative imaging features) of the elastic net*

The correlation matrices of the top ranked clinical benchmark features of the elastic net are shown for each subgroup.

# **S12. Supplementary Figure 10**


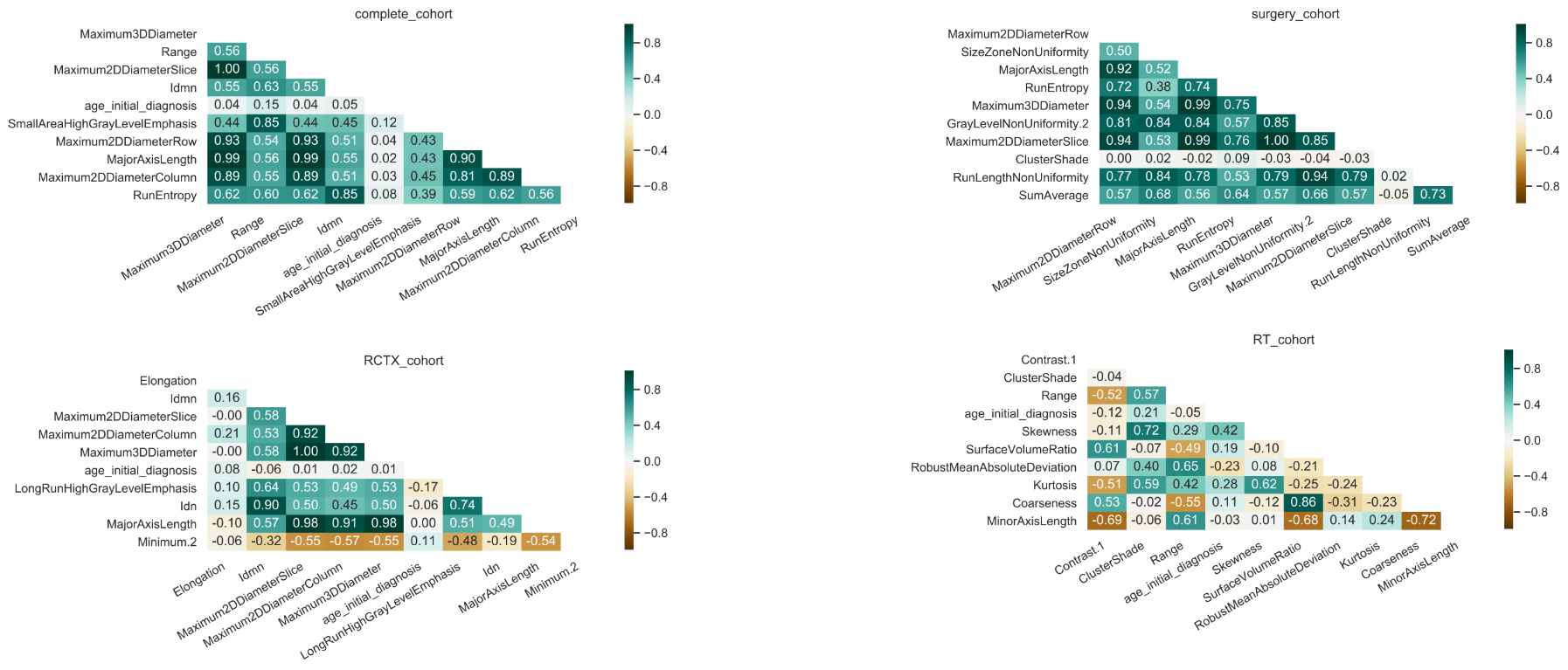


**Supplementary Figure 10:** *Correlation matrix of the top ranked combined features (clinical features and quantitative imaging features) of the random survival forest*

The correlation matrices of the top ranked clinical benchmark features of the random survival forest are shown for each subgroup.

# **S13. Intraclass correlation analysis: radiomics feature classes**


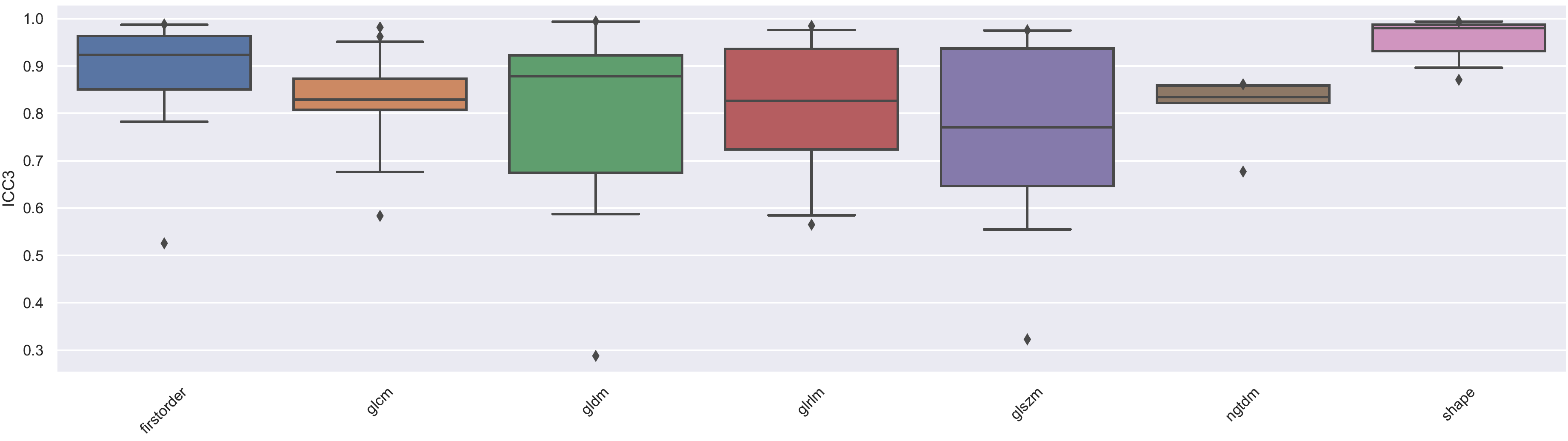


**Supplementary Figure 11:** *Box-Whisker Plots depicting the intraclass correlation analysis for each radiomic feature class.*

**Supplementary Table 1**

| ICC3 | mean | std |
| --- | --- | --- |
| firstorder | 0.892765 | 0.111505 |
| glcm | 0.829713 | 0.091283 |
| gldm | 0.789568 | 0.198878 |
| glrlm | 0.811247 | 0.139182 |
| glszm | 0.766598 | 0.186472 |
| ngtdm | 0.810753 | 0.076467 |
| shape | 0.959575 | 0.040659 |

Intraclass correlation coefficient for each radiomic feature class.

# **S14. Intraclass correlation analysis: individual radiomics features**


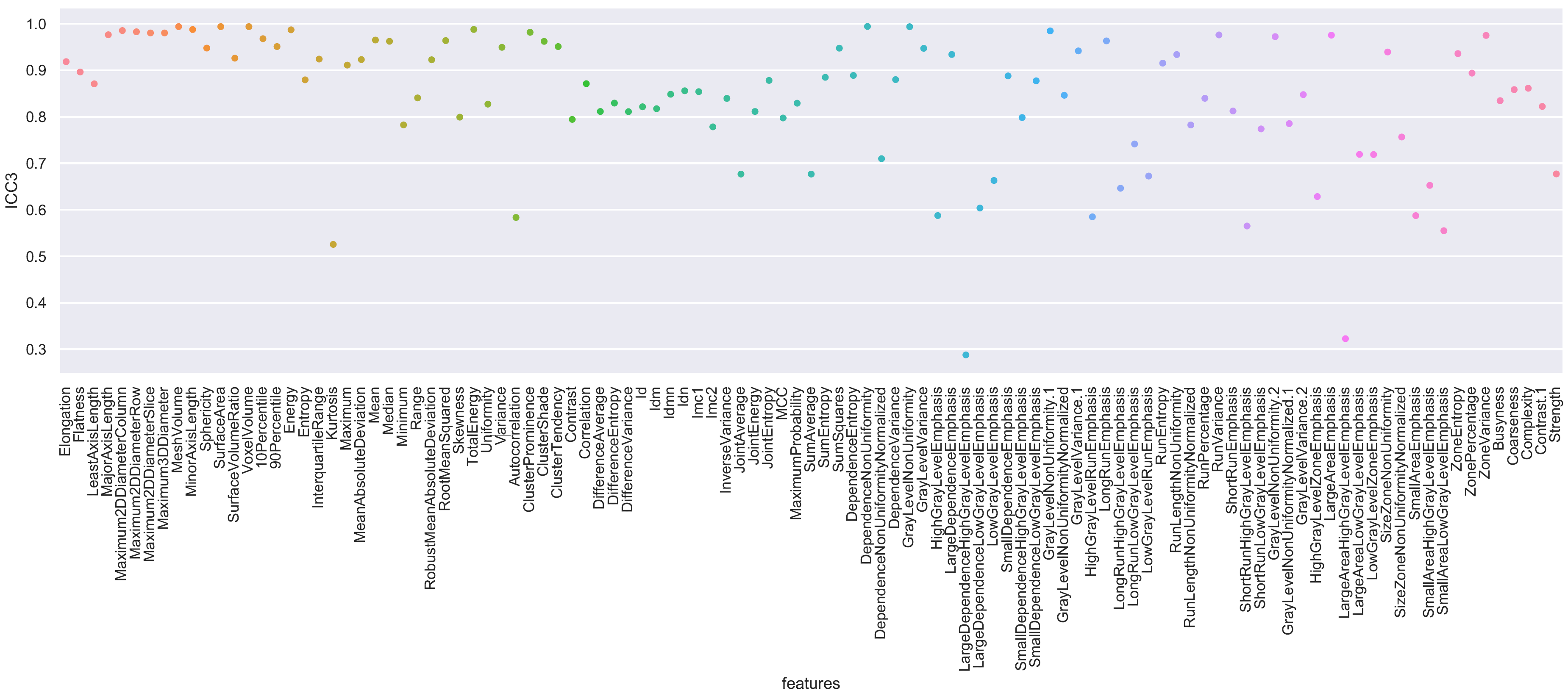


**Supplementary Figure 13:** *Swarmplot depicting the intraclass correlation analysis for each radiomics feature*

**Supplementary Table 2**

| class | features | ICC3 |
| --- | --- | --- |
| shape | Elongation | 0.918549986150725 |
| shape | Flatness | 0.896311921012947 |
| shape | LeastAxisLength | 0.870857704621584 |
| shape | MajorAxisLength | 0.976391092752616 |
| shape | Maximum2DDiameterColumn | 0.985435906853375 |
| shape | Maximum2DDiameterRow | 0.982803110895757 |
| shape | Maximum2DDiameterSlice | 0.980324290547122 |
| shape | Maximum3DDiameter | 0.980300925186512 |
| shape | MeshVolume | 0.993891074486044 |
| shape | MinorAxisLength | 0.987594307708676 |
| shape | Sphericity | 0.94772978472873 |
| shape | SurfaceArea | 0.993895720634783 |
| shape | SurfaceVolumeRatio | 0.926048839597811 |
| shape | VoxelVolume | 0.993910382986245 |
| firstorder | 10Percentile | 0.96791013754633 |
| firstorder | 90Percentile | 0.951055214901 |
| firstorder | Energy | 0.987035394950269 |
| firstorder | Entropy | 0.879470572186315 |
| firstorder | InterquartileRange | 0.924111673744584 |
| firstorder | Kurtosis | 0.52555426544439 |
| firstorder | Maximum | 0.91114106107782 |
| firstorder | MeanAbsoluteDeviation | 0.923007144678946 |
| firstorder | Mean | 0.965057140429168 |
| firstorder | Median | 0.962302177559765 |
| firstorder | Minimum | 0.782504078685851 |
| firstorder | Range | 0.840615458517847 |
| firstorder | RobustMeanAbsoluteDeviation | 0.922588626885064 |
| firstorder | RootMeanSquared | 0.963808938506727 |
| firstorder | Skewness | 0.799196024132732 |
| firstorder | TotalEnergy | 0.987864156915085 |
| firstorder | Uniformity | 0.827173338028575 |
| firstorder | Variance | 0.949375021685611 |
| glcm | Autocorrelation | 0.583504242442002 |
| glcm | ClusterProminence | 0.981671783611314 |
| glcm | ClusterShade | 0.962202466512774 |
| glcm | ClusterTendency | 0.950993054531809 |
| glcm | Contrast | 0.794388629736959 |
| glcm | Correlation | 0.871182096922918 |
| glcm | DifferenceAverage | 0.811400965321331 |
| glcm | DifferenceEntropy | 0.829454885747104 |
| glcm | DifferenceVariance | 0.811021189751608 |
| glcm | Id | 0.821512078137515 |
| glcm | Idm | 0.817391109746915 |
| glcm | Idmn | 0.848439594993971 |
| glcm | Idn | 0.855922320740388 |
| glcm | Imc1 | 0.854006835890445 |
| glcm | Imc2 | 0.778337728355124 |
| glcm | InverseVariance | 0.839484329006216 |
| glcm | JointAverage | 0.676770178770745 |
| glcm | JointEnergy | 0.811386096335467 |
| glcm | JointEntropy | 0.878201076261528 |
| glcm | MCC | 0.797423798979568 |
| glcm | MaximumProbability | 0.829221480363476 |
| glcm | SumAverage | 0.676770178770745 |
| glcm | SumEntropy | 0.884886562045969 |
| glcm | SumSquares | 0.947544994942894 |
| gldm | DependenceEntropy | 0.888928647747006 |
| gldm | DependenceNonUniformity | 0.994279068930569 |
| gldm | DependenceNonUniformityNormalized | 0.709907984217914 |
| gldm | DependenceVariance | 0.87995504688516 |
| gldm | GrayLevelNonUniformity | 0.993686289253771 |
| gldm | GrayLevelVariance | 0.947255262950827 |
| gldm | HighGrayLevelEmphasis | 0.587584686259994 |
| gldm | LargeDependenceEmphasis | 0.934049413843903 |
| gldm | **LargeDependenceHighGrayLevelEmphasis** | 0.287884707644572 |
| gldm | LargeDependenceLowGrayLevelEmphasis | 0.603765536336119 |
| gldm | LowGrayLevelEmphasis | 0.66301714802953 |
| gldm | SmallDependenceEmphasis | 0.887945498729703 |
| gldm | SmallDependenceHighGrayLevelEmphasis | 0.798320622388655 |
| gldm | SmallDependenceLowGrayLevelEmphasis | 0.877368304415474 |
| glrlm | GrayLevelNonUniformity.1 | 0.984652303026298 |
| glrlm | GrayLevelNonUniformityNormalized | 0.846295142998368 |
| glrlm | GrayLevelVariance.1 | 0.94168971277409 |
| glrlm | HighGrayLevelRunEmphasis | 0.584896099979054 |
| glrlm | LongRunEmphasis | 0.963207284887106 |
| glrlm | LongRunHighGrayLevelEmphasis | 0.646360264056176 |
| glrlm | LongRunLowGrayLevelEmphasis | 0.741482690888776 |
| glrlm | LowGrayLevelRunEmphasis | 0.672531994897764 |
| glrlm | RunEntropy | 0.915370985189996 |
| glrlm | RunLengthNonUniformity | 0.933795876791962 |
| glrlm | RunLengthNonUniformityNormalized | 0.782398710878869 |
| glrlm | RunPercentage | 0.839741581582455 |
| glrlm | RunVariance | 0.976038160682358 |
| glrlm | ShortRunEmphasis | 0.812476005785097 |
| glrlm | ShortRunHighGrayLevelEmphasis | 0.565117796124033 |
| glrlm | ShortRunLowGrayLevelEmphasis | 0.773899211654495 |
| glszm | GrayLevelNonUniformity.2 | 0.972365017228419 |
| glszm | GrayLevelNonUniformityNormalized.1 | 0.785304999175962 |
| glszm | GrayLevelVariance.2 | 0.847666038545824 |
| glszm | HighGrayLevelZoneEmphasis | 0.628428355639501 |
| glszm | LargeAreaEmphasis | 0.975495396209845 |
| glszm | **LargeAreaHighGrayLevelEmphasis** | 0.322836436639412 |
| glszm | LargeAreaLowGrayLevelEmphasis | 0.719161147679617 |
| glszm | LowGrayLevelZoneEmphasis | 0.718798604018803 |
| glszm | SizeZoneNonUniformity | 0.939332161955271 |
| glszm | SizeZoneNonUniformityNormalized | 0.756523734796768 |
| glszm | SmallAreaEmphasis | 0.587412486354166 |
| glszm | SmallAreaHighGrayLevelEmphasis | 0.652521019091791 |
| glszm | SmallAreaLowGrayLevelEmphasis | 0.554979301896908 |
| glszm | ZoneEntropy | 0.935880411789534 |
| glszm | ZonePercentage | 0.893875409137885 |
| glszm | ZoneVariance | 0.974988746694906 |
| ngtdm | Busyness | 0.834639053854299 |
| ngtdm | Coarseness | 0.858396496738861 |
| ngtdm | Complexity | 0.861381959592283 |
| ngtdm | Contrast.1 | 0.822211329548122 |
| ngtdm | Strength | 0.677135923354763 |

Intraclass correlation coefficient for each radiomics feature. 2 features revealed an ICC < 0.4 (poor), marked in bold.
